# Early vs. Late Oral Feeding After Surgery for Patients with Esophageal Malignancy: A Systematic Review and Meta-Analysis of Postoperative Clinical Outcomes and Quality of Life

**DOI:** 10.3390/jpm15070317

**Published:** 2025-07-15

**Authors:** Raghad Fahad Alrasheed, Abdullah Salem Laradhi, Reema Saeed Alqahtani, Sarah Mazin Alharthi, Waleed Amin Alamoudi, Zainudheen Faroog, Sham Yasser Almohammad, Jana Ayman Basmaih, Nasser Turki Alotaibi, Ahmed Elaraby, Raed A. Albar, Ayman M. A. Mohamed

**Affiliations:** 1 College of Medicine, Alfaisal University, Riyadh 11533, Saudi Arabia; rfalrasheed@alfaisal.edu (R.F.A.); alaradhi@alfaisal.edu (A.S.L.); reealqahtani@alfaisal.edu (R.S.A.); smalharthi@alfaisal.edu (S.M.A.); walamoudi@alfaisal.edu (W.A.A.); zfaroog@alfaisal.edu (Z.F.); salmohammad@alfaisal.edu (S.Y.A.); jbasmaih@alfaisal.edu (J.A.B.); nalotaibi03@alfaisal.edu (N.T.A.); ralbar@alfaisal.edu (R.A.A.); 2 Faculty of Medicine, Al-Azhar University, 11884 Cairo, Egypt; aelarabymd@gmail.com

**Keywords:** early oral feeding, esophageal cancer, esophagectomy, anastomotic leakage

## Abstract

**Introduction:** Esophagectomy for esophageal cancer traditionally involves delayed postoperative oral feeding due to concerns about complications like anastomotic leakage. Enhanced Recovery After Surgery (ERAS) protocols favor early oral feeding (EOF), but its safety and benefits remain debated. This systematic review and meta-analysis compared EOF versus late oral feeding (LOF) after esophagectomy. **Methods:** We systematically searched PubMed, Scopus, Web of Science, EMBASE, and the Cochrane Library through March 2025 for primary studies comparing EOF (≤7 days post-op) with LOF (>7 days or delayed) in adult patients after esophagectomy. Outcomes included anastomotic leakage, pneumonia, other complications, gastrointestinal recovery, length of hospital stay (LOS), quality of life (QoL), and mortality. **Results:** Twenty-nine studies involving 3962 patients were included. There was no significant difference in the risk of anastomotic leakage between the two groups (RR: 1.03, 95% CI: 0.80–1.33, *p* = 0.82, I^2^ = 0%). EOF was associated with a significantly shorter time to first flatus (Cohen’s d: −1.26, 95% CI: −1.93 to −0.58, *p* < 0.001) and first defecation (Cohen’s d: −0.87, 95% CI: −1.51 to −0.22, *p* = 0.01) and a shorter LOS (*p* = 0.01). No significant differences were found for other complications (acute respiratory distress syndrome [ARDS], chyle leak, conduit issues, ileus, sepsis, wound infection) or mortality rates (in-hospital, 30-day, 90-day, overall). QoL assessment suggested potential improvement in emotional function with EOF. **Conclusions:** EOF after esophagectomy appears safe, as it does not increase the risk of anastomotic leakage or other major complications compared to LOF. It is associated with faster gastrointestinal recovery and shorter hospital stays, supporting its use within ERAS protocols.

## 1. Introduction

Esophageal cancer is a significant health concern, ranking as the eighth most common cancer worldwide and the sixth leading cause of cancer-related mortality worldwide [1].

The cornerstone of curative treatment for resectable esophageal cancer is esophagectomy, often in conjunction with neoadjuvant therapy [2,3]. For locally advanced disease, the standard of care involves neoadjuvant therapy to improve oncological outcomes. Depending on tumor histology and location, this most commonly includes perioperative chemotherapy (such as the FLOT regimen) or neoadjuvant chemoradiotherapy (such as the CROSS regimen) [4,5].

While immunotherapy has become a standard in the adjuvant setting for certain patient populations and is crucial for treating advanced or metastatic disease, the optimal neoadjuvant strategies continue to evolve, with ongoing trials investigating novel combinations [5,6].

Surgical resection, whether minimally invasive or open, involves the removal of part or all of the esophagus, with reconstruction typically using a portion of the stomach to create a gastric conduit [7,8]. Minimally invasive esophagectomy (MIE) has emerged as a safe and durable technique and is increasingly adopted. Also, different surgical approaches are still valid with good outcomes, including the Ivor Lewis and McKeown techniques [9,10].

Traditionally, postoperative management following esophagectomy has involved a period of nil-by-mouth for several days, often for at least five days, with nutritional support provided via enteral tube feeding (e.g., nasogastric or nasojejunal tubes) distal to the new anastomosis. This practice was historically driven by the fear of postoperative complications, particularly aspiration pneumonia, anastomotic leakage, and their sequelae [10]. Surgeons have been reluctant to start oral intake early due to perceived risks and a lack of high-quality evidence from clinical trials [10].

However, the concept of Enhanced Recovery After Surgery (ERAS) protocols has gained significant traction in various types of major abdominal surgery, including esophagectomy. ERAS protocols aim to reduce postoperative morbidity and improve recovery through a multimodal approach encompassing various perioperative interventions. The early start of oral intake is an essential component of ERAS protocols [11,12,13].

Studies have shown the feasibility of allowing food at will in major upper gastrointestinal surgery without increasing postoperative morbidity [11,14,15]. Emerging evidence suggests potential benefits of early oral feeding following esophagectomy, including faster recovery, improved recovery of intestinal function, shorter length of hospital stay, and enhanced patient satisfaction and quality of life. Some studies indicate that early oral feeding does not lead to an increased rate or severity of anastomotic leakage or aspiration pneumonia in selected patients [8,10]. Existing studies on early oral feeding after esophagectomy have sometimes involved highly selected patient populations, different surgical techniques (including non-standard reconstructions), and variations in the timing and methods of early oral intake [9,10,16].

Based on the ongoing debate and the potential advantages of early oral feeding, our meta-analysis aims to systematically review the available evidence comparing early and late oral feeding after surgery for patients with esophageal malignancy. Our review will focus on evaluating the impact of early oral feeding on postoperative quality of life and clinical outcomes such as the incidence of complications (e.g., anastomotic leakage and pneumonia), length of hospital stay, and mortality.

## 2. Methods

We followed the Preferred Reporting Items for Systematic reviews and Meta-Analyses (PRISMA) statement guidelines when performing this systematic review and meta-analysis. The method was carried out in accordance with the Cochrane handbook of systematic reviews and meta-analysis of interventions version 5.1.0 and the AMSTAR-2 (Assessing the Methodological Quality of Systematic Reviews 2) Guidelines. We prospectively registered the protocol for this meta-analysis on PROSPERO (CRD420251017918).

### 2.1. Eligibility Criteria

We considered all English primary studies involving adult patients (aged 18 years or older) who have undergone any type of surgery for esophageal or esophagogastric cancer and were treated following early oral feeding protocols. These studies should compare early oral feeding to late or traditional oral feeding protocols, where oral intake is delayed beyond the predefined early oral feeding timelines. Late or traditional feeding protocols are often supplemented by tubal or enteral nutrition methods, such as jejunostomy or intravenous (IV) supplementation.

Studies must report at least one of the following outcomes: postoperative complications (e.g., anastomotic leakage, pneumonia, or wound infection), nutritional status and caloric intake, length of hospital stay, time to return of bowel function (e.g., time to first flatus or defecation), mortality rates, or quality of life score assessments.

We further excluded studies involving pediatric patients or those who did not receive the intervention, as well as studies that do not clearly define the timing of oral feeding initiation post-esophagectomy, lack relevant outcome data related to postoperative recovery, complications, or nutritional status, or are non-comparative in nature. We also excluded case reports, reviews, letters to the editor, conference abstracts, and non-English publications.

### 2.2. Literature Search

We performed a comprehensive literature search across five electronic databases (PubMed, Scopus, Web of Science, EMBASE, and the Cochrane Library) from their inception to March 2025, using suitable keywords. The search strategies were adjusted for each database, and no language or publication restrictions were applied. The full strategy for each database is illustrated in Supplementary Table S1. All duplicate references were removed by Zotero (version 6.0, https://www.zotero.org).

### 2.3. Screening of the Literature Search Results

The literature search results were screened in a two-step process. Initially, the titles and abstracts of all articles were assessed for eligibility. We used the Rayyan website. After identifying relevant records, full-text screening was conducted for the studies that met the eligibility criteria. We performed a manual backward and forward analysis for citations of the included studies. The screening was conducted by applying the predefined inclusion criteria.

### 2.4. Study Arms and Definitions

We included studies that compared two postoperative nutritional strategies: EOF and LOF. EOF was defined as the initiation of oral intake, either gradually or in combination with supplemental nutrition (e.g., enteral or parenteral support), within ≤7 days following surgery, irrespective of diet type. LOF was characterized by delayed oral feeding initiation until clinical confirmation of gastrointestinal recovery, such as the presence of bowel sounds or flatus. Patients in the LOF group adhered to traditional nil-by-mouth (NPO) practices, relying exclusively on enteral or parenteral nutrition until gastrointestinal safety was established.

Our main outcomes were postoperative complications (e.g., anastomotic leakage, pneumonia, wound infection), nutritional status, length of hospital stay, and mortality rates.

### 2.5. Data Extraction

Data from the included studies was extracted and recorded in a standardized data extraction sheet. The extracted data encompassed four main categories: (1) characteristics of the included studies, (2) characteristics of the study population, (3) risk of bias domains, and (4) outcome measures.

### 2.6. Synthesis of Results

For outcomes that involved dichotomous data, the frequency of events and the total number of patients in each group were combined to calculate the relative risk using the DerSimonian–Laird random-effects model. For outcomes that involved continuous data, such as mean difference (MD) and standard deviation (SD), the DerSimonian–Laird random-effects model was used to combine the results. In cases where studies reported data multiple times, the last point of follow-up was considered for the primary analysis. All statistical analyses were conducted using STATA 18 software.

### 2.7. Assessment of Heterogeneity

The presence of statistical heterogeneity among studies was assessed using the Cochrane Q test, which calculates the chi-square statistic (Q). The I-squared (I2) statistic was then calculated using the formula I^2^ = ((Q − df)/Q) × 100%. A *p*-value of less than 0.1 for the chi-square test was considered as indicative of significant heterogeneity, and I-squared values equal to or greater than 50% were considered as indicative of high heterogeneity.

### 2.8. Quality Assessment and Publication Bias

Three authors independently evaluated the quality of the included clinical trials using the Cochrane Risk of Bias 2 tool for RCTs, which involves assessing five domains: randomization process (selection bias), deviation from intended interventions (performance bias), outcome measurement (detection bias), missing outcome data (attrition bias), selection of reported results (reporting bias), and other potential sources of bias. The authors’ assessment decisions were categorized as ‘Low risk of bias’, ‘High risk of bias’, or ‘Some concerns’. Any discrepancies among the three authors were resolved through discussion with a fourth author.

Observational quality was assessed using the Newcastle–Ottawa Scale (NOS). The included studies were evaluated based on three main domains: selection of study participants, comparability of study groups, and the ascertainment of outcomes for cohort studies or the exposure for case-control studies. Each domain consists of several sub-domains, with a maximum score of 9 points indicating the highest quality. Included studies scoring 7 or more were considered high quality, while those scoring below 7 were classified as high-risk of bias studies.

To investigate the possibility of publication bias among the studies, we created funnel plots that present the relationship between effect size and standard error. We used two methods to assess evidence of publication bias: (1) Egger’s regression test and (2) the Begg and Mazumdar rank correlation test (Kendall’s tau).

We evaluated the overall quality of our evidence using the Grading of Recommendations, Assessment, Development, and Evaluation (GRADE) system, which uses four levels of quality: high, moderate, low, and very low. The GRADE approach contains several domains, including study design, risk of bias, inconsistency, indirectness, imprecision, publication bias, effect size, dose–response relationships, and all residual confounding factors.

### 2.9. Certainty Assessment

We performed a certainty assessment using sensitivity analysis, also known as leave-one-out meta-analysis, to evaluate the robustness of the evidence. For each outcome included in the meta-analysis, we conducted a sensitivity analysis in various scenarios by excluding one study at a time to ensure that the overall effect size was not heavily influenced by any single study.

## 3. Results

### 3.1. Results of the Literature Search and Screening

**Figure 1:**
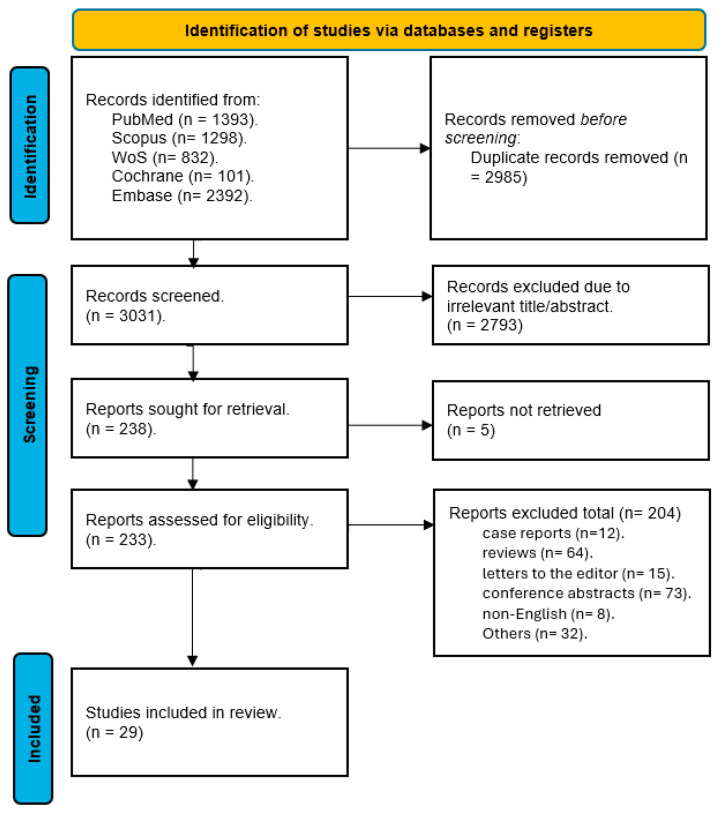
PRISMA flow diagram for studies’ selection process [17].

We conducted a comprehensive literature search across the five major databases: PubMed, Scopus, Web of Science, Cochrane CENTRAL, and EMBASE. The initial search yielded a total of 6016 records. After removing duplicates and irrelevant records, we screened 3031 unique studies for eligibility based on their titles and abstracts. We excluded 2793 studies that did not meet our inclusion criteria. We obtained the full-text articles of the remaining 238 studies for further assessment. After applying the predefined criteria, we excluded 209 studies for various reasons, such as lack of relevant outcomes, inappropriate study design, or poor quality. Finally, we included 29 studies in our systematic review and meta-analysis. The flow diagram of the literature search and screening process is shown in Figure 1.

### 3.2. Characteristics of Included Studies and Risk of Bias Assessment

Our systematic review included 29 studies comparing EOF and LOF after surgery for esophageal malignancy, with a total of 3962 patients (Table 1). The definition of EOF varied but generally involved initiating oral intake within the first 7 postoperative days (PODs), often starting as early as POD 0 or 1 with liquids and gradually progressing. LOF typically involved delaying oral intake until at least POD 5–7 or later, often relying on nil-by-mouth (NPO) status with enteral tube feeding (e.g., nasojejunal or jejunostomy) or parenteral nutrition in the interim.

Our populations consisted of adults (≥18 years) undergoing various types of esophagectomy (e.g., minimally invasive esophagectomy [MIE], transhiatal esophagectomy [THE], minimally invasive McKeown esophagectomy [MIMCE], or Ivor Lewis) for esophageal or esophagogastric cancer, primarily esophageal squamous cell carcinoma (ESCC) or esophageal adenocarcinoma (EAC). Baseline characteristics such as age (typically in the 60s), sex (predominantly male), BMI, comorbidities, tumor location/stage, and prior treatments are reported in Table 2.

**Figure 2:**
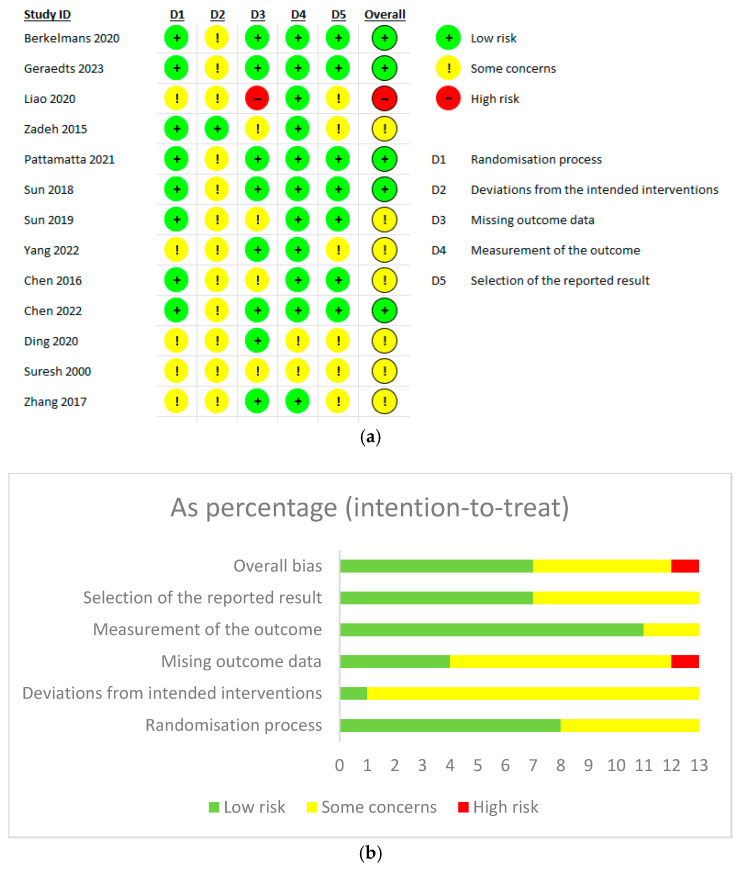
(**a**) Cochrane Risk of Bias Assessment tool 2 for randomized control trials. (**b**) Intention-to-treat analysis [8,10,11,20,21,22,23,24,29,30,31,38,40].

The quality of included RCTs was assessed using the Cochrane Risk of Bias 2 tool, and we found that most of our included studies showed an overall unclear risk of bias. Five studies showed an overall low risk of bias, while one study was judged to have a high risk of bias due to a marked proportion of missing outcome data that exceeded five percent, without the application of intention-to-treat analysis (Figure 2a,b).

The quality of observational studies was evaluated using NOS, and we found most studies were of good overall quality. Two studies were rated as moderate quality. One study had a particularly poor score in the comparability domain, as it failed to report any baseline characteristic comparisons between groups. Participant selection was consistently rated as high quality across all included studies. Although five studies received low scores in the outcome domain, only two of these were classified as having moderate overall quality (Supplementary Table S2).

### 3.3. Anastomosis Leakage

**Figure 3:**
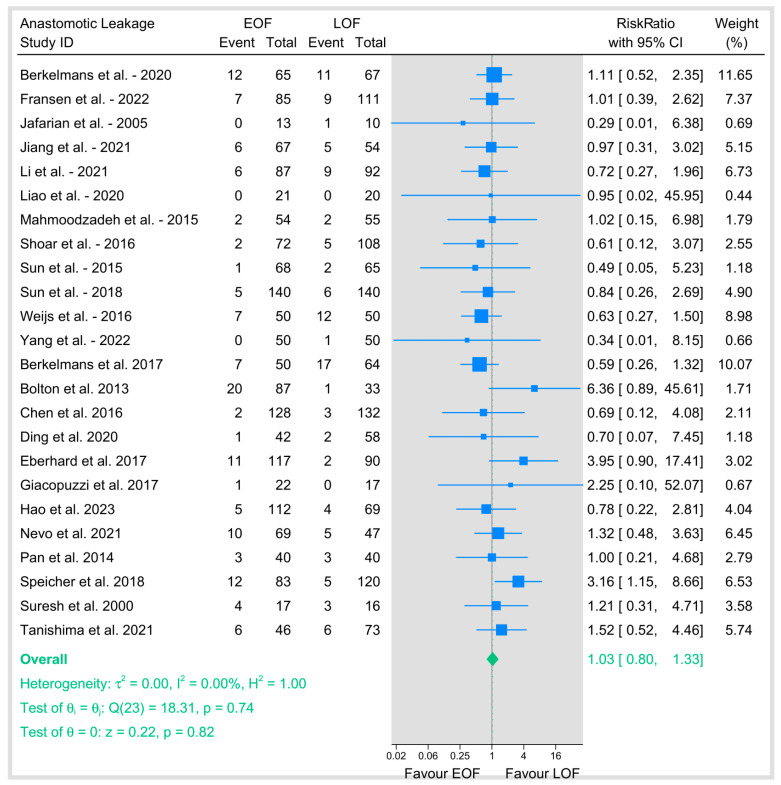
Assessment of anastomotic leakage [8,9,10,12,16,18,19,20,21,23,25,26,27,28,29,31,32,33,34,35,36,37,38,39].

Our primary outcome of anastomotic leakage was evaluated across 24 studies, involving a total of 3166 patients (1585 in the EOF group and 1581 in the LOF group). The pooled risk ratio (RR) was 1.03 (95% CI: 0.8–1.33; *p* = 0.82), indicating no significant difference between the two feeding strategies (Figure 3). Heterogeneity was negligible (I^2^ = 0%, *p* = 0.74), suggesting consistency across studies.

We conducted a subgroup analysis based on the surgical approach. In minimally invasive esophagectomy (MIE), thirteen studies showed a pooled RR of 0.89 (95% CI: 0.64–1.22; *p* = 0.46) with no heterogeneity (I^2^ = 0%, *p* = 0.98). Open surgery showed a pooled RR of 1.34 (95% CI: 0.7–2.58; *p* = 0.38) with moderate heterogeneity (I^2^ = 31.23%, *p* = 0.22).

**Figure 4:**
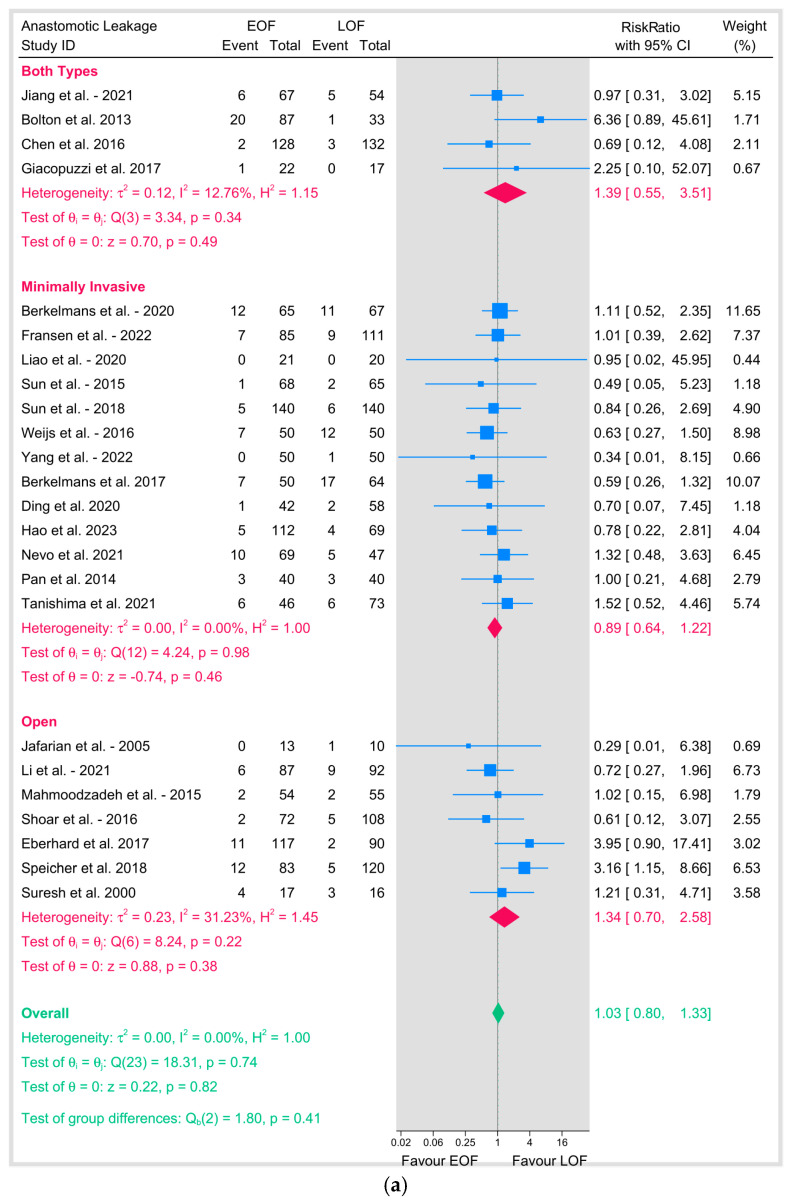

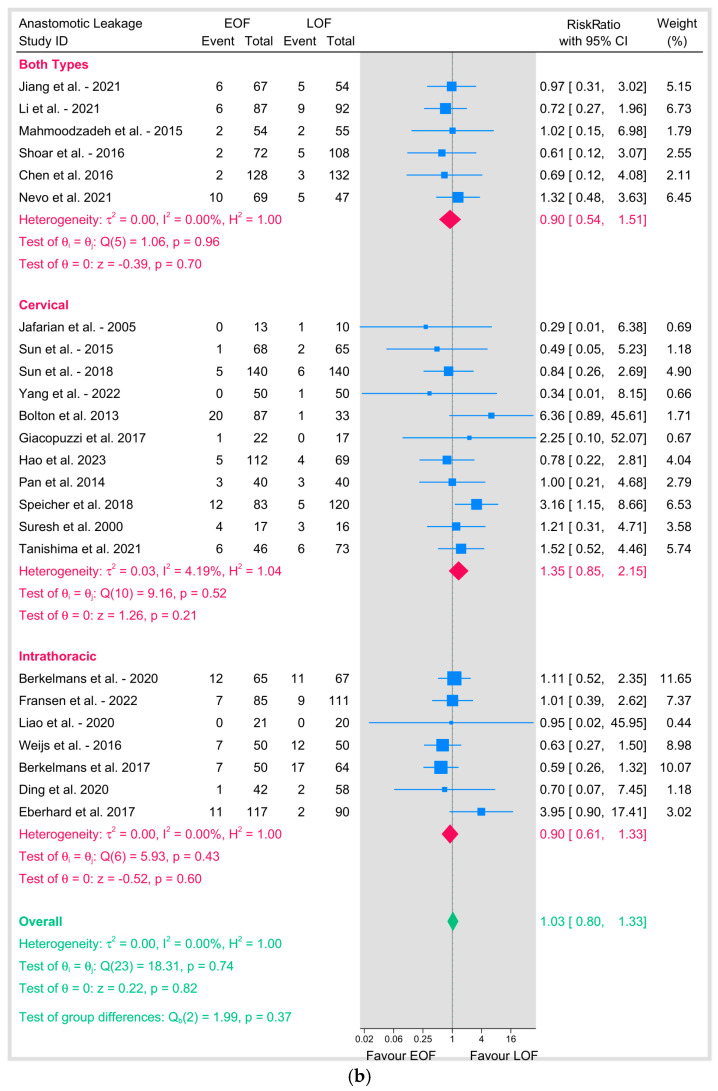
(**a**) Anastomotic leakage based on surgical technique. (**b**) Anastomotic leakage based on position of anastomosis [8,9,10,12,16,18,19,20,21,23,25,26,27,28,29,31,32,33,34,35,36,37,38,39].

Studies that included both types of surgery showed a pooled RR of 1.39 (95% CI: 0.55–3.51; *p* = 0.49) with low heterogeneity (I^2^ = 12.76%, *p* = 0.34), and tests of group differences across surgical approaches was not significant (*p* = 0.41), indicating that the effect of EOF versus LOF on anastomotic leakage did not vary by surgical technique (Figure 4a).

Similarly, subgroup analyses based on the position of the anastomosis showed no significant difference between the two arms either in cervical anastomosis (11 studies; RR = 1.35, 95% CI 0.85–2.15, *p* = 0.2; I^2^ = 4.19%) or intrathoracic anastomosis (7 studies; RR = 0.9, 95% CI 0.61–1.33, *p* = 0.6; I^2^ = 0%) (Figure 4b).

**Figure 5:**
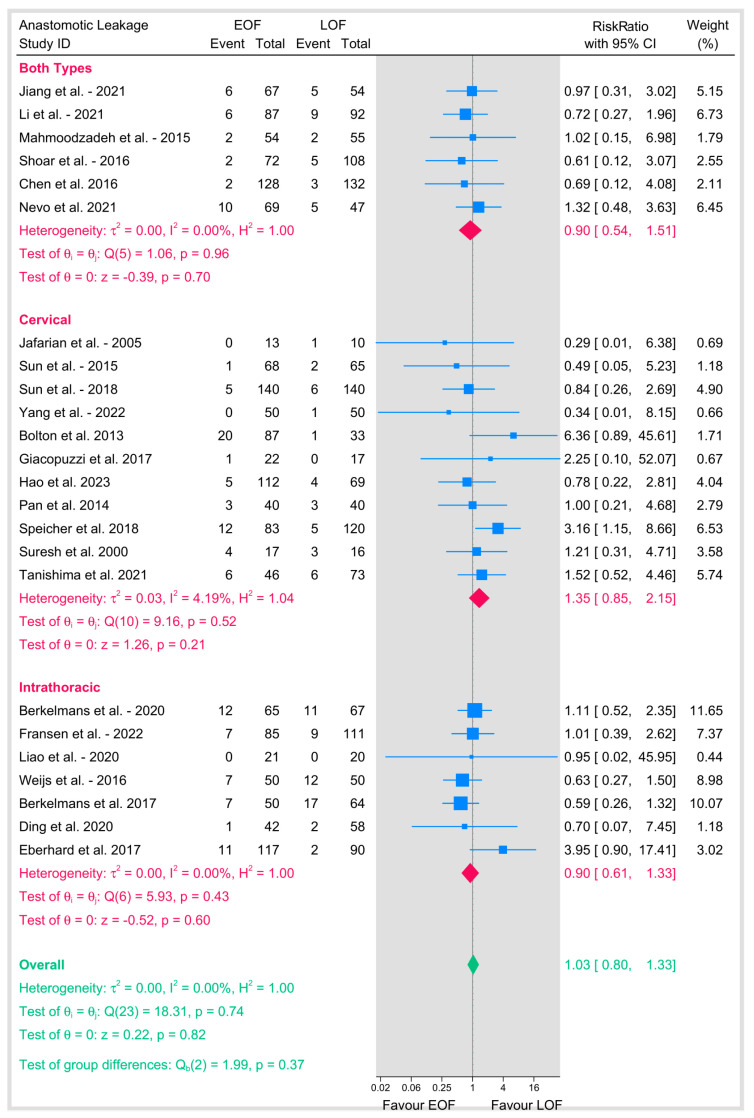
Anastomotic leakage based on time of introduction of oral feeding [8,9,10,12,16,18,19,20,21,23,25,26,27,28,29,31,32,33,34,35,36,37,38,39].

To address potential variations in defining “early,” we did a subgroup analysis by the specific postoperative day (POD) of oral feeding initiation (Figure 5). Initiation on POD 0 (RR 0.9, 95% CI 0.56–1.47), POD 1 (RR 0.9, 95% CI 0.59–1.37), and POD 2 (RR 1.02, 95% CI 0.52–1.96) each demonstrated no significant effect on anastomotic leakage and showed no heterogeneity (I^2^ = 0%). Critically, no subgroup showed a statistically significant increase or decrease in risk. The test for subgroup differences was not significant (*p* = 0.79).

**Figure 6:**
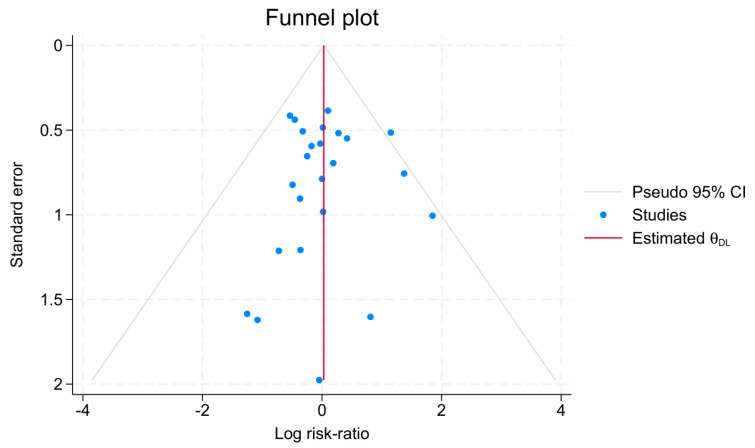
Anastomotic leakage funnel plot assessment of bias.

Publication bias was assessed for anastomotic leakage using Egger’s regression test and the nonparametric trim-and-fill method. Egger’s test showed no evidence of small-study effects (beta1 = 0.05, SE = 0.528, z = 0.10, *p* = 0.923). The trim-and-fill analysis imputed zero studies, with the adjusted RR remaining identical to the observed RR (1.03, 95% CI: 0.796–1.333), indicating no significant publication bias. A funnel plot further supported symmetry and absence of bias (Figure 6).

### 3.4. Respiratory Complications (Figure 7)

#### 3.4.1. Acute Respiratory Distress Syndrome (ARDS)

Four studies evaluated the incidence of ARDS between EOF and LOF, with a pooled RR of 0.75 (95% CI: 0.29–1.91; *p* = 0.54). No heterogeneity was observed (I^2^ = 0%, *p* = 0.86), suggesting no difference in ARDS risk between EOF and LOF.

**Figure 7:**
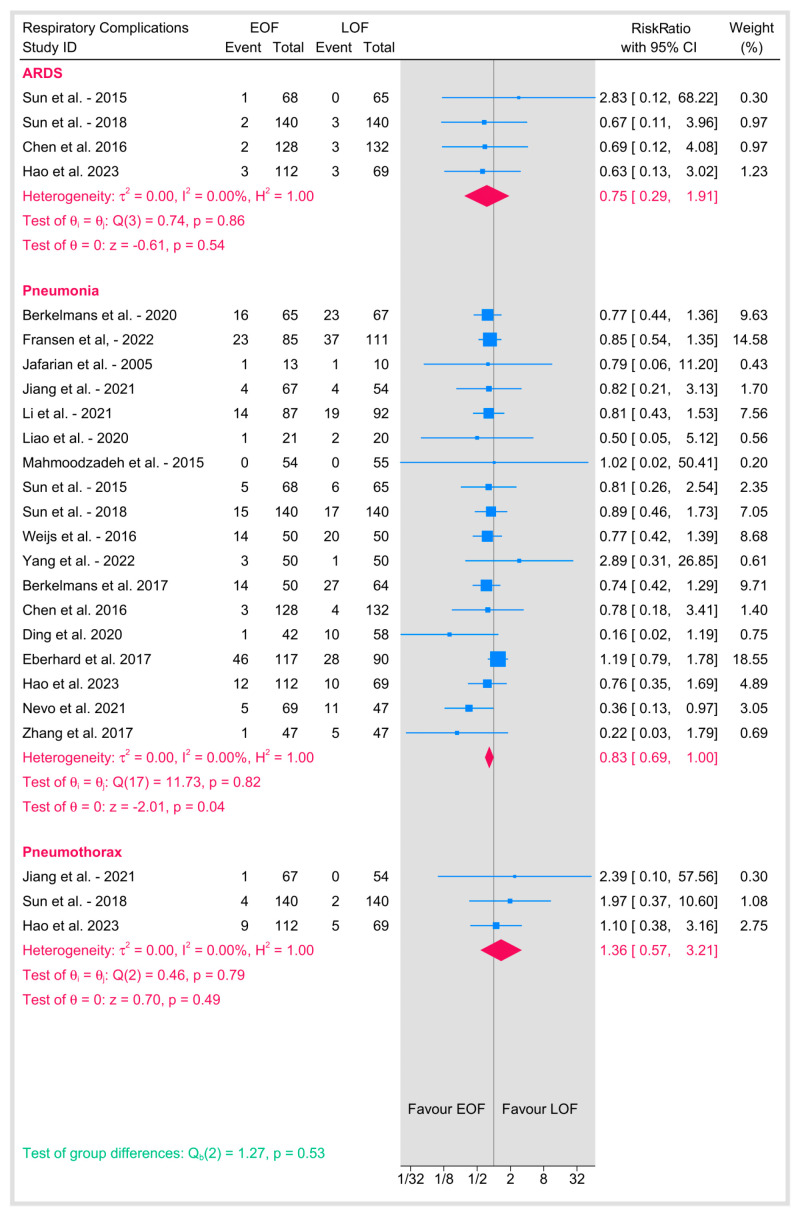
Respiratory complications [8,9,10,12,16,18,19,20,21,23,25,26,27,28,29,31,32,33,34,35,36,37,38,39].

#### 3.4.2. Pneumonia

Seventeen studies reported the incidence of pneumonia, with a pooled RR of 0.83 (95% CI: 0.69–1; *p* = 0.04) with no heterogeneity (I^2^ = 0%), suggesting a minimally significant lower risk of pneumonia among the EOF group.

#### 3.4.3. Pneumothorax

Three studies reported the rate of pneumothorax, with a pooled RR of 1.36 (95% CI: 0.57–3.21; *p* = 0.49). No heterogeneity was observed (I^2^ = 0%, *p* = 0.79). The results suggest no significant difference in pneumothorax risk between EOF and LOF.

#### 3.4.4. Arrhythmias

**Figure 8:**
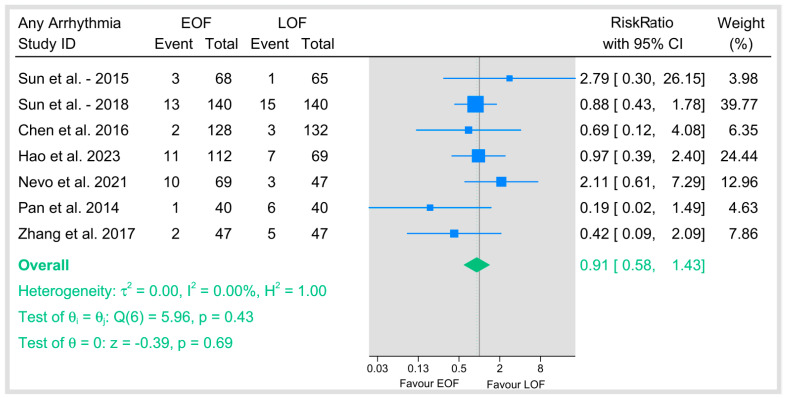
Any arrhythmias [9,23,29,34,35,36,40].

The RR of pooled incidence of arrhythmia is 0.91 (95% CI: 0.58–1.43), with no statistical difference between the EOF and LOF groups (*p* = 0.69). The analysis showed no heterogeneity (I^2^ = 0%) (Figure 8).

#### 3.4.5. Other Postoperative Complications

**Figure 9:**
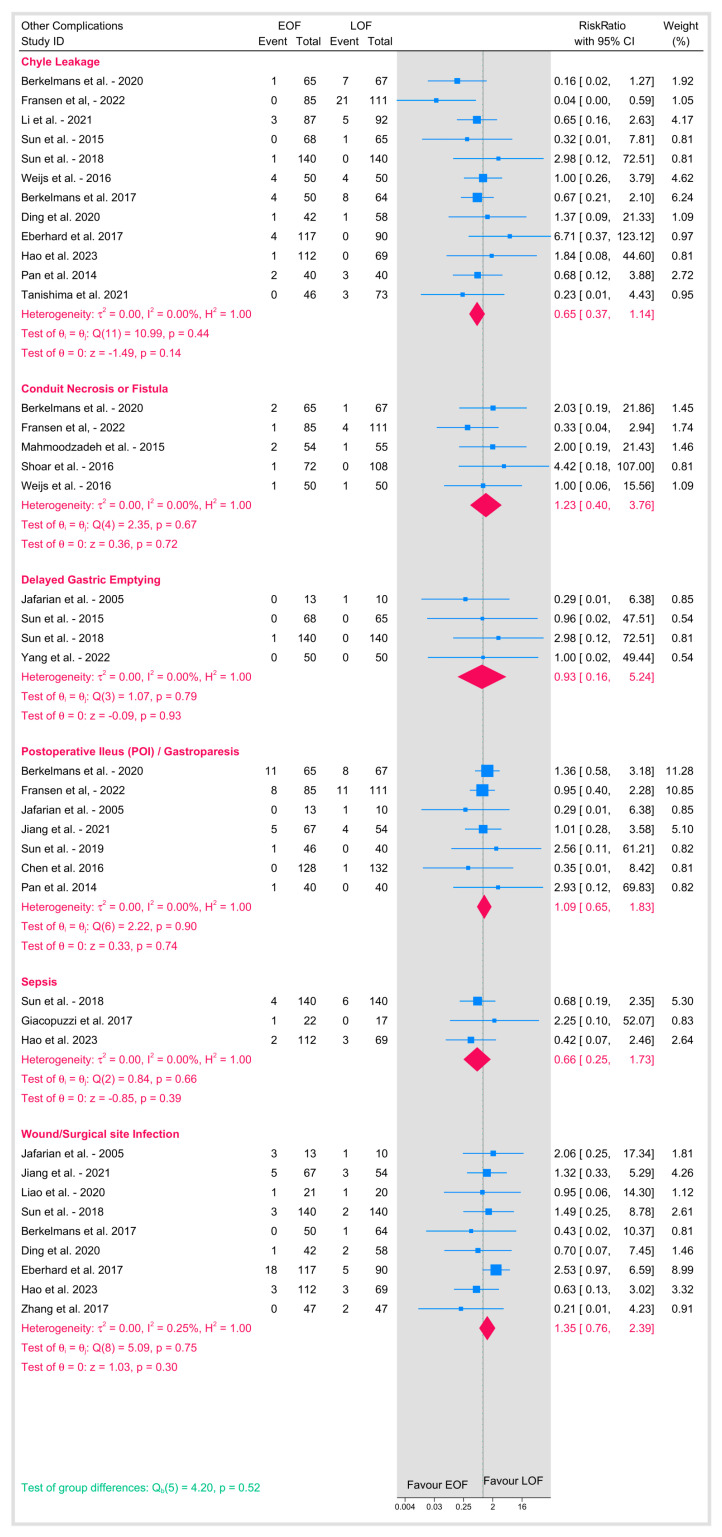
Other postoperative complications [8,9,10,12,16,18,19,20,21,23,25,26,27,28,29,31,32,33,34,35,36,37,38,39].

Additional complications, including chyle leakage, conduit necrosis or fistula formation, delayed gastric emptying, postoperative ileus (or gastroparesis), sepsis, and wound/surgical site infection, were also analyzed (Figure 9). The pooled estimates for these outcomes showed minimal heterogeneity (I^2^ between 0% and 0.25%), and all pooled RR were non-significant for each complication (e.g., chyle leakage: *p* = 0.14; conduit necrosis/fistula: *p* = 0.72; delayed gastric emptying: *p* = 0.93; postoperative ileus: *p* = 0.74; sepsis: *p* = 0.39; wound infections: *p* = 0.30). The overall test for differences across these complications was also not significant (*p* = 0.52), demonstrating that early oral feeding does not significantly alter the risk of these additional postoperative events when compared to late feeding.

### 3.5. Gastrointestinal Recovery Outcomes

#### 3.5.1. Time to First Bowel Movement/Defecation

**Figure 10:**
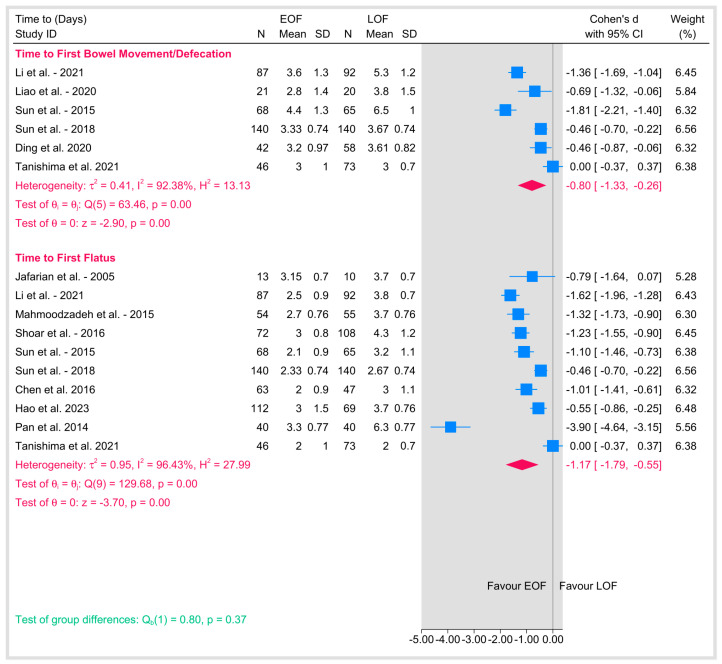
Gastrointestinal recovery outcomes [9,18,19,20,21,23,26,29,31,34,36,39].

Shown the results of the summary of gastrointestinal recovery outcomes with the subgroups in the Figure 10.

Five studies reported on the time to first bowel movement or defecation following esophagectomy. The pooled Cohen’s d was −0.8 (95% CI: −1.33 to −0.26; *p* = 0.01), indicating a moderate to large effect favoring EOF, with a significantly shorter time to first bowel movement compared to LOF. However, substantial heterogeneity that we could not resolve either by sensitivity or subgrouping (I^2^ = 92.38%, *p* < 0.001) suggests differences in study populations, methodologies, or definitions of the endpoint. Individual study effect sizes ranged from −1.81 as shown in Sun et al., 2015 [9] to zero as shown in Tanishima et al., 2021 [39], reflecting differences in the magnitude of the effect of EOF on this outcome.

#### 3.5.2. Time to First Flatus

Similarly, nine studies assessed the time to first flatus as a marker of gastrointestinal recovery. The pooled Cohen’s d was −1.17 (95% CI: −1.79 to −0.55; *p* < 0.001), indicating a large effect favoring EOF, with a significantly shorter time to first flatus compared to LOF. Considerable heterogeneity was present (I^2^ = 96.43%, *p* < 0.001), suggesting significant variability across studies. Individual study effect sizes ranged from −3.90 as shown in Pan et al., 2014 [36] to 0 as shown in Tanishima et al., 2021 [39], highlighting variability in the impact of EOF on this outcome. A test of group differences between time to first bowel movement and time to first flatus showed no significant difference in the effect of EOF versus LOF across these two outcomes (*p* = 0.41).

#### 3.5.3. Length of Hospital Stay

**Figure 11:**
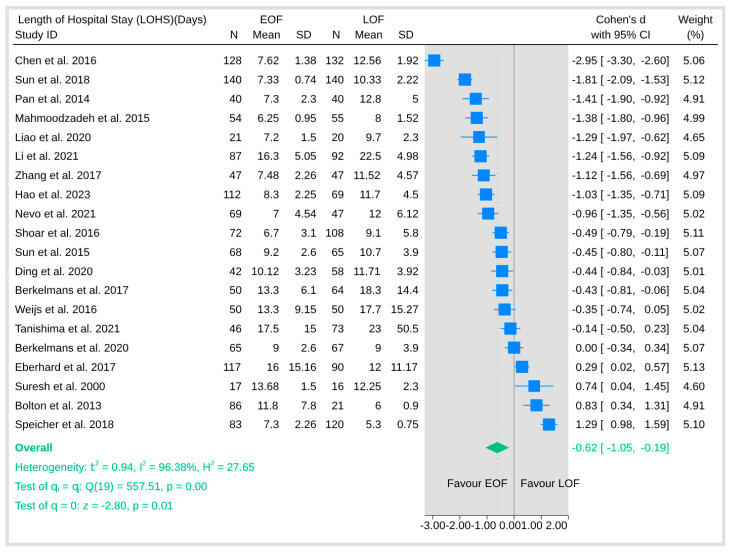
Length of hospital stay [8,9,10,12,16,18,19,20,21,23,25,26,27,28,29,31,32,33,34,35,36,37,38,39].

The pooled analysis of length of hospital stay was associated with a significantly shorter hospitalization duration in the EOF arm (Figure 11). The overall effect (Cohen’s d) was statistically significant (*p* = 0.01). However, the analysis also revealed substantial heterogeneity (I^2^ = 96.38%).

#### 3.5.4. Hospital Readmission Rate

**Figure 12:**
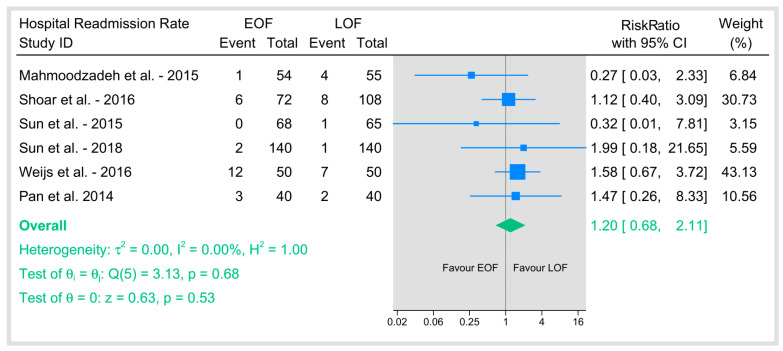
Hospital readmission rate [9,21,23,25,26,36].

The pooled six studies that reported readmission rates revealed no significant difference between the two arms (Figure 12). The pooled risk ratio was not statistically significant (*p* = 0.53), and there was no evidence of heterogeneity among studies (I^2^ = 0%). These findings indicate that EOF does not significantly affect the risk of hospital readmission compared with LOF, supporting the overall safety profile of early oral feeding in postoperative management.

#### 3.5.5. Quality of Life

**Figure 13:**
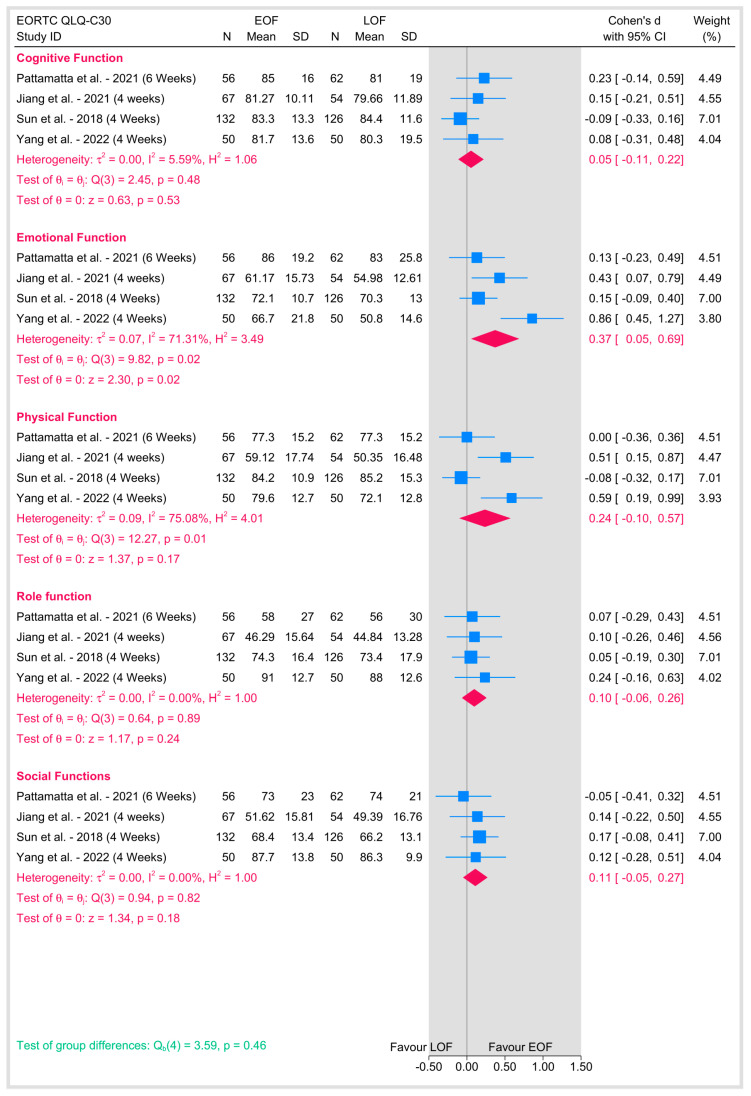
Quality of life [8,16,22,23].

Quality of life was assessed using the EORTC QLQ-C30 questionnaire at 4 to 6 weeks postoperatively in the included studies (Figure 13). In the domain of cognitive function, no significant heterogeneity was observed (I^2^ = 5.59%), and the pooled analysis did not show a statistically significant difference between the two arms (*p* = 0.53). In contrast, emotional function demonstrated moderate-to-high heterogeneity (I^2^ = 71.31%), with the pooled effect favoring EOF reaching statistical significance (*p* = 0.02), suggesting that patients receiving early oral feeding experienced better emotional outcomes compared with those undergoing late oral feeding. For physical function, there was also considerable heterogeneity (I^2^ = 75.08%), but the overall effect did not reach significance (*p* = 0.17), indicating similar physical functioning between groups. Role function and social function, on the other hand, exhibited no heterogeneity (I^2^ = 0% for both) and showed no significant differences between EOF and LOF. Moreover, the overall test for group differences across these quality-of-life domains was not significant (*p* = 0.46), implying that while early oral feeding appears to have a positive impact on emotional well-being, its effects on other quality-of-life domains remain comparable to those observed with late feeding.

#### 3.5.6. Mortality

**Figure 14:**
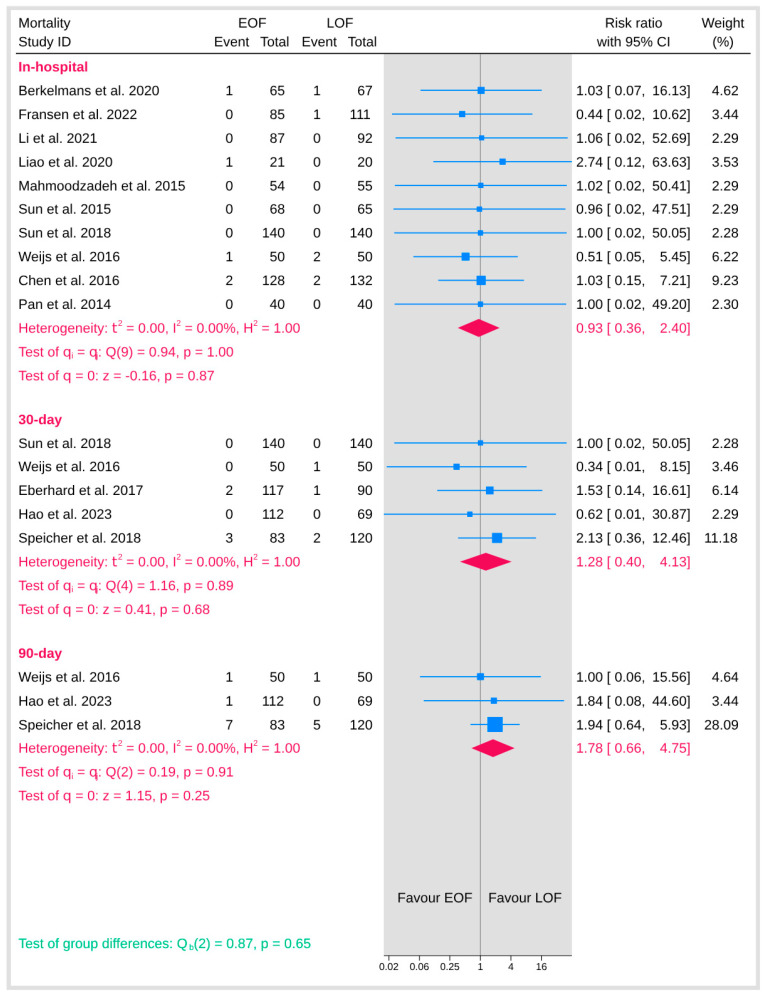
Mortality [9,10,12,19,20,21,23,25,29,32,34,36,37].

Mortality outcomes were evaluated across multiple time points, including in-hospital, 30-day, 90-day, and overall mortality (Figure 14). For each of these endpoints, there was no detectable heterogeneity (I^2^ = 0%), indicating consistent effects across studies. The pooled analysis for in-hospital mortality showed no significant difference between the two arms (*p* = 0.87). Similarly, the analysis for 30-day mortality did not reveal any significant difference (*p* = 0.68), and the 90-day mortality analysis also failed to show a statistically significant effect (*p* = 0.25). In addition, the overall test for group differences across the mortality outcomes was non-significant (*p* = 0.65), suggesting that initiating early oral feeding does not increase the risk of mortality compared with late oral feeding.

#### 3.5.7. Overall Survival at Last Point of Follow-Up

**Figure 15:**
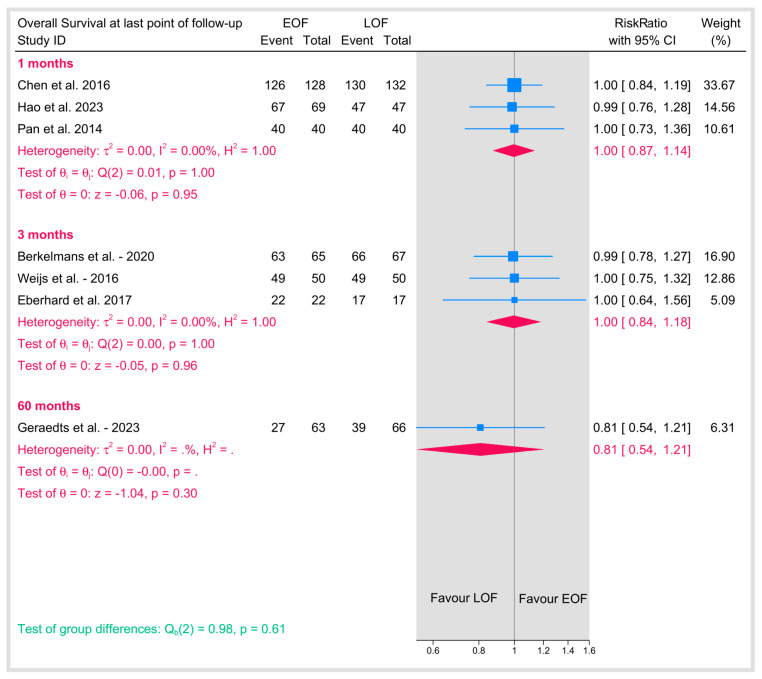
Overall survival at last point of follow-up [10,11,25,29,32,34,36].

Overall survival was evaluated at the final follow-up across studies with varying time points, ranging from 1 month and 3 months to as long as 60 months (Figure 15). The pooled analysis revealed that EOF did not adversely affect overall survival compared with LOF. The pooled risk ratio was approximately 1.00, with tests for heterogeneity indicating no variability among the studies (I^2^ = 0%). These findings support the fact that the timing of postoperative oral feeding does not have a detrimental impact on long-term overall survival in patients.

#### 3.5.8. Quality of Evidence

The quality of evidence for our outcomes was assessed using the GRADE framework (Supplementary Table S3). The evidence regarding anastomotic leakage and 30-day mortality was rated ‘Moderate’. Both outcomes were downgraded from ‘High’ due to serious imprecision, as the confidence intervals for the point estimates crossed the null effect value. The evidence for a reduction in pneumonia was also ‘Moderate’, downgraded for imprecision given the confidence interval’s proximity to the line of no effect.

In contrast, the evidence supporting a shorter time to first flatus, first defecation, and reduced length of hospital stay was rated ‘Low’. This downgrade reflects concerns regarding risk of bias and, more critically, very serious inconsistency (I^2^ > 90%), indicating substantial heterogeneity in the magnitude of the observed benefits across studies.

## 4. Discussion

Our review is the most comprehensive and up-to-date synthesis of data from 29 studies, including 3962 patients, to evaluate the impact of EOF compared to LOF following surgery for esophageal malignancy. The primary finding demonstrated no statistically significant difference in the incidence of the postoperative complication of anastomotic leakage between the EOF and LOF cohorts.

In addition, EOF did not increase the risk of various other significant complications, including ARDS, pneumothorax, chyle leakage, conduit necrosis/fistula, sepsis, or wound infections, nor did it adversely affect in-hospital, 30-day, 90-day, or overall long-term mortality. On the other hand, the start of EOF was associated with statistically significant benefits in accelerating gastrointestinal recovery, evidenced by shortened times to first flatus and first defecation. Consistent with enhanced recovery principles, EOF also correlated with a reduced length of hospital stay and showed a modest reduction in postoperative pneumonia rates.

Quality of life assessments suggested an improvement in emotional function with EOF, although other domains showed no significant difference; however, this should be interpreted with caution. The finding of a potential improvement in emotional function is based on a small pool of only five studies that employed heterogeneous assessment tools and follow-up schedules. Furthermore, because we analyzed multiple QoL sub-scales simultaneously, there is an inherent risk of a Type I error (a false-positive result). Therefore, this finding on emotional function should be considered exploratory and hypothesis-generating rather than conclusive evidence of a benefit. Our finding that EOF does not significantly increase the risk of anastomotic leakage is the most clinically impactful result, directly addressing the historical concern that has driven traditional nil-by-mouth protocols post-esophagectomy. The pooled risk ratio is approximately the same, alongside minimal heterogeneity across a large dataset (24 studies). This outcome aligns with several recent RCTs and observational studies included in this synthesis [10,23], as well as meta-analyses focusing on upper gastrointestinal surgery [41,42]. Also, subgroup analyses stratified by both surgical approach (MIE vs. open) and anastomotic site (cervical vs. thoracic) failed to show a differential effect, suggesting the safety of EOF regarding leakage may be generalizable across common surgical variations. While some older studies [28,32,37] (e.g., Bolton et al., 2013; Eberhard et al., 2017; and Speicher et al., 2018 which found potential benefit with delay) were included, the overall weight of evidence, particularly from recent prospective trials, supports the conclusion that early introduction of oral nutrition does not compromise anastomotic integrity.

A valid criticism of our results is the significant heterogeneity in the definition of “early” oral feeding. Our initial analysis grouped studies with EOF initiation from POD 0 to POD 7. Our results revealed that no single start day—from the day of surgery (POD 0) through the first week—was associated with a statistically significant increase in anastomotic leakage risk. The non-significant group differences further reinforce the conclusion that the safety of EOF concerning anastomotic integrity appears consistent across different “early” start times. This directly addresses the clinical concern that very early feeding (e.g., POD 0–2) might be riskier than a slightly later, yet still early, start.

Furthermore, it was questioned whether the observed benefits in gastrointestinal recovery—a reduction of over one day for first flatus and nearly one day for first defecation—are clinically meaningful. We contend that in the context of ERAS protocols, such improvements are indeed significant. An accelerated return of bowel function is a key determinant for patient comfort, tolerance of nutrition, and readiness for discharge. When combined with the demonstrated reduction in length of hospital stay, these benefits represent improvements in patient recovery and a more efficient use of healthcare resources.

Bolton et al. and Speicher et al. focused on patients undergoing transhiatal esophagectomy (THE) with a cervical esophagogastric anastomosis (CEGA), which carries a higher intrinsic risk of leakage compared to intrathoracic anastomoses [28,37]. Eberhard et al., who studied mostly Ivor Lewis procedures (intrathoracic anastomosis), found the lowest leak rate in their most conservative regimen (delayed start with extended blended diet), suggesting that even within potentially lower-risk anastomoses, extreme caution might yield benefits, although their standard delayed group had intermediate results [32].

Another critical difference is the definition and the duration of the “late oral feeding” intervention. Our meta-analysis likely employed a common threshold (e.g., initiation > POD 7) to categorize studies. However, the protocols described by Speicher et al. (delay until POD 15) and Bolton et al. (mean delay to POD 12) represent more prolonged periods of NPO than might be typical in other studies classified as “late” (e.g., starting POD 8 or 9) [28,37]. It is possible that the duration of anastomotic rest is the key determinant of protective efficacy; a 12- to 15-day delay will also allow for more wound healing, collagen deposition, and revascularization compared to shorter delays. The reduction in leak rates reported in these studies may be caused by the extended NPO periods, which is a benefit not generalized to all protocols simply falling beyond the POD 7 cutoff.

Eberhard’s most protective regimen further complicates this, combining a POD 7 NPO period with an extended slow progression using a blended diet until POD 21, suggesting that dietary consistency and progression speed, not just initial delay, are also variables [32].

By pooling some studies with widely varying LOF durations and progression strategies under a single “late” category, our meta-analysis might hide the specific benefits associated with these more conservative or prolonged protocols.

Our significant acceleration of gastrointestinal recovery results, manifested as reduced time to first flatus and defecation, is consistent with established physiological principles where early enteral stimulation promotes gut motility by hormonal pathways such as incretins and direct mechanical effects [43,44].

The mechanism for the potential reduction in pneumonia (borderline significance, RR 0.83, *p* = 0.04), while requiring cautious interpretation, might relate to several factors facilitated by EOF. Enhanced patient mobilization, due to reduced interventions with tubes and intravenous lines, will potentially improve respiratory mechanics and possibly avoid the complications sometimes associated with nasoenteral tube feeding.

The improvement noted in emotional function postoperatively is also important; avoiding the psychological burden associated with prolonged fasting and tube feeding likely contributes positively to the patient’s overall well-being and recovery. These combined benefits strongly favor EOF’s role for esophageal cancer patients.

Comprehensive safety assessment across numerous postoperative adverse events still favors EOF. The lack of significant difference in the rates of ARDS, pneumothorax, chyle leakage, conduit complications, delayed gastric emptying, ileus, sepsis, and wound infections suggests that even the stresses induced by postoperative feeding are not contributors to these complications compared to an LOF strategy, which typically involves alternative nutritional support routes. Also, the consistent finding of no increased mortality risk at any assessed time point (in-hospital, 30-day, 90-day, overall survival) provides more reassurance.

This comprehensive safety profile, coupled with the recovery benefits, strongly challenges the rationale for routine prolonged postoperative fasting following esophagectomy in most patients. Our results demonstrating accelerated gastrointestinal recovery and a shorter length of hospital stay are highly consistent with this evidence, establishing these as reliable benefits of EOF. This study advances the field in several key aspects. First, with a dataset comprising 29 studies and nearly 4000 patients, our analysis represents the most updated and statistically robust synthesis to date, providing greater precision for critical safety outcomes. Second, unlike some earlier reviews that were hesitant to pool leakage data due to conflicting results from older retrospective studies, our inclusion of recent, high-quality RCTs allows for a more conclusive quantitative assessment.

For heterogeneity in our results, despite the clear findings for several key outcomes like anastomotic leakage and mortality (where I^2^ was negligible), significant heterogeneity was observed for variables related to the speed of recovery, time to first flatus (I^2^ = 96.14%), time to first bowel movement/defecation (I^2^ = 92.01%), and length of hospital stay (I^2^ = 96.12%), as well as for certain quality-of-life domains (emotional and physical function).

We believe that the contributing factors probably include variations in the specific EOF protocols implemented (e.g., initiation day ranging from POD 0/1 to PODs 5–7 within the broad “early” definition, differing diet types, and progression speeds) and the comparator LOF protocols (varying NPO duration and supplemental nutrition methods).

Additionally, differences in patient populations (baseline nutritional status, comorbidities, and neoadjuvant treatment exposure), surgical techniques (the specifics of MIE/open approaches and anastomosis methods), center volume/experience, definitions of outcomes (especially subjective bowel function timing), and discharge criteria (impacting LOS) are all contributors to this observed heterogeneity. While sensitivity analyses did not specify any single outlying study, this high degree of heterogeneity necessitates caution in interpretation.

Our finding regarding anastomotic leakage aligns strongly with the most recent comprehensive meta-analyses by Mei et al. (2022) [41] and Hong et al. (2023) [45], which also concluded that EOF does not significantly increase the risk of this complication. This marks a consolidation of evidence compared to earlier reviews like Liu et al. (2019) [46] and Zhang et al. (2020) [47], which pointed to conflicting retrospective studies and refrained from pooling leakage data due to heterogeneity. By pooling a substantial number of the most recent studies, our current analysis provides comprehensive quantitative support for the safety of EOF concerning anastomotic procedures following esophagectomy.

Regarding postoperative recovery and patient survival, our results are highly consistent with those reported by Mei et al. and Hong et al. [41,45]. We demonstrated that EOF significantly increases recovery, including reduced time to first flatus and defecation, and leads to a shorter length of hospital stay. Our analysis also found no association between EOF and increased mortality rates. This consistent evidence across multiple reviews points to the EOF’s dual advantages, which are enhancing recovery and maintaining safety.

For most other secondary postoperative complications, such as chyle leakage, our findings are generally similar to previous studies such as Hong et al., indicating no significant differences between early and late feeding [45]. We observed a borderline statistical trend towards lower pneumonia rates with EOF; this differs from the pneumonia results by Mei et al. and Hong et al., which is a minor difference between our review and their results that we believe will not alter the overall favorable safety of EOF across the spectrum of common complications.

Our evidence challenges the rationale for routine prolonged postoperative fasting. Adopting EOF can potentially improve emotional well-being, reduce healthcare resource utilization, and may offer a modest benefit regarding pulmonary complications. While the optimal EOF protocol requires further definition due to observed heterogeneity in our results, we believe that EOF should be considered for further assessment as the default postoperative nutritional pathway to gain conclusive evidence.

## 5. Strengths and Limitations

Our strengths include the inclusion of the most substantial and up-to-date studies involving a large patient number, affording considerable statistical power. We evaluated a broad spectrum of clinically relevant outcomes, including complications, recovery indicators, mortality, quality of life, and survival, providing a comprehensive assessment.

Having said that, the interpretation of our findings must consider certain limitations. Substantial heterogeneity, especially for recovery speed metrics and some quality-of-life domains, points to variability likely arising from diverse EOF/LOF protocols, patient populations, and surgical techniques. Another limitation is the exclusion of non-English language publications. However, we expect that the overall impact on our primary conclusions to be minimal, as most large-scale, international trial studies are published in English. Also, our meta-analysis includes a mix of randomized controlled trials and observational studies. While this broadens the available evidence, the inherent potential for selection bias and confounding in non-randomized studies could influence the results.

In addition, our quality assessment revealed that many included RCTs had “some concerns” for bias, particularly related to the randomization process or deviation from intended interventions. While the observational studies were generally of good quality, they are inherently at a higher risk of confounding. This mix of evidence, dominated by observational data, is still a key limitation.

For future research, we suggest large-scale, multicenter RCTs utilizing standardized EOF protocols—specifying initiation timing, diet composition, and progression—compared to equally well-defined control regimens and comparative effectiveness studies evaluating different EOF strategies (e.g., POD 1 vs. POD 3; liquid vs. soft start).

The safety profile and accelerated recovery benefits identified in this meta-analysis provide a strong clinical guide to establishing EOF as the standard of care within ERAS protocols for esophagectomy. Our evidence suggests that routine prolonged late feeding is often unnecessary and may be delaying recovery and negatively impacting the patient experience. The safety and accelerated recovery benefits of EOF support its use as part of the standard of care after esophagectomy, but individualized protocols that take into account individual patient differences are needed. Clinical judgment remains paramount, and a risk-stratified strategy is likely optimal. Patients with high-risk features, such as a tenuous anastomosis, significant comorbidities, or poor preoperative nutritional status, may still benefit from a more cautious, modified EOF protocol. Future research should focus on refining these protocols, comparing different EOF strategies (e.g., timing of initiation, diet composition, and progression), and identifying patient subgroups that benefit most, thereby moving from the question of whether we should feed early to how we can best implement early feeding for all.

## 6. Conclusions

Our systematic review of nearly 4000 patients provides strong evidence that EOF following esophagectomy does not increase the risk of the most feared complication, anastomotic leakage, compared to traditional LOF. EOF also did not demonstrate an increased risk for other significant postoperative complications.

Our analysis highlights significant benefits associated with EOF, primarily a faster return of gastrointestinal function (earlier flatus and defecation) and a reduction in the overall length of hospital stay. Quality of life assessments showed mixed results with improvement of emotional function. These findings challenge the traditional practice of prolonged nil-by-mouth status after esophagectomy.

## Figures and Tables

**Table 1:** Summary of included studies.

Study ID	Study Design	Study Setting	Country	Total Sample Size	Study Arms	Definition of the Intervention Arm	Time of Feeding	Definition of the Comparator Arm	Name of Procedure	Definition of Patients’ Disease	List of Reported Outcomes	Last Point of Follow-Up
Berkelmans 2020 [10]	RCT	Multicenter	Netherlands, Sweden	132	EOF vs. LOF	POD 0: Immediate initiation of oral feeding after surgery	POD 0	POD 0–5: NPO with tube feeding POD 5: Initiation of oral feeding	MIE with intrathoracic anastomosis	Patients with esophageal cancer	Time to functional recovery, anastomotic leakage, pneumonia, other complications	1 month
Fransen et al., 2022 [12]	Prospective Cohort	Single-center	Netherlands	196	DOF vs. SOC	POD 0: Sips of water (up to 250 cc) POD 1: Liquid food (up to 500 cc) POD 5: Liquid food (up to 1500 cc) POD 15: Solid food	POD 0	POD 0–5: NPO with tube feeding and sips of water (up to 250 cc) POD 5: Initiation of oral feeding POD 20: Solid food	MIE with intrathoracic anastomosis	-	Time to functional recovery, length of hospital stay, anastomotic leakage, pneumonia, chyle leakage, cardiopulmonary complications, jejunostomy complications, postoperative ileus, 30-day postoperative complication rate	1 month
Geraedts 2023 [11]	RCT	Multicenter	Netherlands, Sweden	145	EOF vs. LOF	POD 0: Sips of water (up to 250 cc) POD 1: Liquid food (up to 500 cc) POD 5: Liquid food (up to 1500 cc)	POD 0	POD 0–5: Sips of water (up to 250 cc) POD 5: Start oral feeding	MIE-IL	Patients with esophageal cancer	3-year and 7-year overall survival, disease-free survival, impact of complications and caloric intake on overall survival	60 months
Jafarian 2005 [18]	Prospective cohort	Single-center	Iran	23	EOF vs. SOC	POD 2: NG tube removed POD 3: Liquid food POD 4–9: Soft regular diet	POD 3	POD 4–5: NG tube removed POD 5–7: Initiation of oral feeding	THE with cervical esophagogastrostomy	Patients with esophageal cancer	Nausea, vomiting, reinsertion of NG tube, resumption of appetite, anastomotic leakage, wound infection, pulmonary problems, weight loss	-
Jiang 2021 [16]	Retrospective cohort	Single-center	China	121	EOF vs. LOF	POD 7: Oral feeding	POD 7	POD 7: Initiation of oral feeding	Radical resection of esophageal carcinoma	Patients with ESCC and type II diabetes, without severe comorbidities	Blood glucose levels, complications, nutritional indexes (Hb, ALB, PA), immune indexes (IgA, IgG, IgM), inflammatory indexes (CRP, IL-6), T12-SMA variation, QLQ-C30 scores	1 month
Li 2021 [19]	Retrospective cohort	Single-center	China	179	EOF vs. tube feeding	POD 0–1: NG tube removed POD 0–2: Oral feeding	POD 2	POD 2–5: NG tube removed POD 7: NJ tube removed after confirming no complications	Esophagectomy (MIE-IL, MIMCE, open Ivor Lewis, open McKeown)	Patients with esophageal cancer, without severe cardiac or pulmonary dysfunction	Nutritional status (Hb, ALB, PA), functional outcomes (time to first flatus and defecation), length of ICU stay, length of hospitalization, hospital charges, complications (severe pneumonia, anastomotic leakage, chyle leakage)	Until hospital discharge
Liao 2020 [20]	RCT	Single-center	China	41	EOF vs. LOF	POD 1: 5% glucose in normal saline in addition to tube feeding with EN POD 4: Liquid food	POD 1	POD 0–7: Tube feeding with EN POD 7: Liquid diet	MIE-IL	Patients aged 18–70 with esophageal cancer in the middle or lower esophagus	Nutritional status (ALB, PA), clinical outcomes (complications, mortality, length of hospital stay, time to first oral intake, time to ambulation, bowel movement recovery), inflammatory marker (CRP), incidence of thirst	Until hospital discharge
Mahmoodzadeh 2015 [21]	RCT	Multicenter	Iran	109	EOF vs. LOF	POD 1: 100 mL of tea supplemented with 20 g sugar, gradually increased to 250 mL	POD 1	NPO until bowel sounds return and resolution of ileus	TTE and gastrectomy	Adults (≥18 years) with esophageal or gastric malignancies undergoing surgical resection, stable postoperatively	Length of hospital stay, postoperative complications, intravenous serum volume, NG tube use duration, time to gas passage, post-feeding vomiting, repeated NG tube insertion, time to decrease in serum infusion	Until hospital discharge
Pattamatta 2021 [22]	RCT	Multicenter	Netherlands, Sweden	132	DOF vs. SOC	POD 0: Immediate liquid/purified diet after surgery	POD 0	POD 0–5: NPO with tube feeding	MIE with intrathoracic anastomosis	Adults (≥18 years) with esophageal cancer	Quality of life (EORTC QLQ-C30, QLQ-OG25), tube feeding duration, need for home-care assistance, hospital costs, home-care costs	6 months
Sun 2015 [9]	Cohort	Single-center	China	133	EOF vs. LOF	POD 1: Liquid food	POD 1	POD 7: Liquid food	Thoraco-laparoscopic esophagectomy	Patients with histologically proven ESCC, age < 80 years, adequate organ function	Gastric emptying time, total complications, anastomotic leak, pneumonia, time to first flatus, time to first bowel movement, length of postoperative stay, readmittance within 3 weeks	Until hospital discharge
Sun 2018 [23]	RCT	Single-center	China	280	EOF vs. LOF	POD 1: Liquid food, progressing to soft solids	POD 1	POD 0–7: NPO with tube feeding POD 7: Initiation of oral feeding	MIMCE	Patients with esophageal cancer	Complications, bowel function, QoL, Length of hospital stay, dietary intake	6 months
Sun 2019 [24]	RCT	Single-center	China	86	EOF vs. LOF	POD 1: Liquid food, progressing to soft solids	POD 1	POD 0–7: NPO with tube feeding POD 1: EN via an NJ tube	MIMCE	Patients with esophageal cancer	Inflammatory cytokine levels (IL-6, IL-8, TNF-α, MCP-1), postoperative complications (cardiac, respiratory, gastrointestinal, other).	1 month
Weijs 2016 [25]	prospective cohort	Multicenter	Netherlands	100	EOF vs. LOF	POD 0: Clear liquid food POD 1–6: Liquid food POD 7: Solid food	POD 0	POD 0–4 or POD 0–7: NPO with tube feeding	MIE-IL	Adults (≥18 years) with esophageal cancer	Clinical outcomes (anastomotic leakage, pneumonia, complications, hospital stay, ICU stay, mortality) and nutritional outcomes	3 months
Yang 2022 [8]	RCT	Single-center	China	100	EOF vs. LOF	POD 1: EOF	POD 1	POD 0–7: NPO with tube feeding POD 7: Initiation of oral intake	Laparo-thoracoscopy combined with radical dissection of esophageal cancer, with Jiang’s anastomosis technique	Patients first diagnosed with esophageal cancer without serious cardiopulmonary disease	Six-minute walk test, QLQ-C30, QLQ-OES18, complications (anastomotic leakage, stenosis, delayed gastric emptying, pulmonary infection)	1 month
Shoar 2016 [26]	Prospective Cohort	Single-center	Iran	180	EOF vs. LOF	POD 1–2: Liquid regimen (50 cc black tea and 8 g sugar)	POD 1–2	POD 5: Initiation of oral feeding	Surgical resection for upper gastrointestinal malignancies	Patients with a diagnosis of esophageal or gastric malignancies	Time to tube removal, time to starting soft diet, duration of hospital stay, surgical complications (anastomosis leakage, peritonitis, abscess formation, fistula), rehospitalization, vomiting after initiation of oral feeding	-
Berkelmans 2017 [27]	Retrospective cohort	Multicenter	Netherlands	114	EOF vs. LOF	POD 1–5: Liquid food POD 5: Normal solid diet	POD 1	POD 1: EN via a jejunostomy catheter or an NJ tube POD 5: Initiation of oral feeding	MIE-IL	Adults (≥18 years) with esophageal cancer	Outcome measures include BMI at various stages, weight loss, nutritional re-interventions, postoperative complications, jejunostomy tube-related issues, length of stay, and one-year survival	12 months
Bolton 2013 [28]	Retrospective cohort	Single-center	USA	120	EOF vs LOF	Oral diet delayed until a median of 12 days postoperatively after esophagectomy	POD 5–7	POD 6: Initiation of oral feeding	Esophagectomy with CEGA	Patients with esophageal cancer	Length of stay, delayed gastric emptying, recurrent laryngeal nerve	-
Chen 2016 [29]	RCT	Single-center	China	276	FTS vs. SOC	POD 1: Early enteral nutrition via jejunostomy tube and early mobilization	POD 4	POD 2: NJ feeding	Esophagectomy	Patients aged ≥18 and ≤75 years, with ASA grade I/II, BMI 18.5–27.5 kg/m^2^, and resectable esophageal cancer	Postoperative hospital stay, hospitalization expenditure, postoperative complications (including atrial arrhythmia, ileus, pneumonia, anastomotic leak, incision infection, ARDS), mortality, 30-day readmission rate, incision pain scale (NRS), serum CRP levels, serum globulin levels	1 month
Chen 2022 [30]	RCT	Single-center	China	110	EOF vs. LOF	POD 1–3: Liquid food POD 3: Normal solid diet POD 0–5: Supplementary parenteral nutrition	POD 1	POD 1: EN or an NJ tube POD 1–3: Supplementary parenteral nutrition POD 7: Initiation of oral feeding	MIMCE	Adults (≥18 years) with esophageal cancer	Perioperative serum BGP levels (gastrin, motilin, substance-P, cholecystokinin, somatostatin) measured preoperatively and on PODs 1, 3, and 5, time to first flatus, time to first defecation	-
Ding 2020 [31]	RCT	Single-center	China	100	Observation vs. control	POD 0: EN combined with ERAS	POD 1	POD 1–2: EN combined with IV nutrition POD 7: Oral food intake, progressing from liquid to semi-liquid food under nutritionist guidance POD 7–14: IV nutrition stopped	Ivor Lewis	Patients aged 18–75 years with esophageal cancer indicated malnutrition or nutritional risk	Operation time, intraoperative blood loss, postoperative chest tube removal time, oral feeding time after operation, serum ALB, serum TF, serum PA	Until hospital discharge
Eberhard 2017 [32]	Retrospective cohort	Single-center	Denmark	359	EOF vs. LOF	NPO until POD 7, then normal diet vs. NPO until POD 1, then clear fluids vs. NPO until POD 7, then clear fluids	POD 1	-	Esophagectomy (Ivor Lewis or robot-assisted hybrid with open-thorax technique for GEJ resection)	Adults (≥18 years) with EAC or high-grade dysplasia (Barrett’s esophagus) of the GEJ, without signs of dissemination on preoperative imaging/laparoscopy	Anastomotic leakage (incidence, grade based on modified Urschel/Dindo classification, time to leakage), complications (overall incidence, specific types: pulmonary, cardiac, chylous leakage, wound infection)	2 months
Giacopuzzi 2017 [33]	Prospective cohort	Single-center	Italy	39	ERAS vs. SOC	POD 1: Clear fluids POD 4: Soft diet	POD 1	POD 8: Initiation of soft diet	Esophagectomy (including Ivor Lewis, McKeown, and esophagogastrostomy variations)	Patients with ESCC or EAC Siewert type I or II	Time to extubation; time to ICU discharge; time to removal of drain, urinary catheter, thoracic epidural catheter, and central venous catheter; time to mobilization; time to resume oral intake; length of hospital stay	3 months
Hao 2023 [34]	Retrospective cohort	Single-center	China	181	EOF vs. LOF		POD 1	POD 1: NG feeding and parenteral nutrition provided POD 7: Initiation of oral feeding	MIMCE with Li’s anastomosis	Patients with newly diagnosed, resectable primary esophageal cancer who received neoadjuvant chemotherapy	Postoperative complications	3 months
Nevo 2021 [35]	Retrospective cohort	Single-center	Canada	175	SOC vs. Initial ERP vs. Most Recent ERP	POD 1: Foley catheter removal POD 2: NG tube removal and sips of water, progressing to fluids POD 6: Planned discharge	POD 1	POD 0–3: NG feeding POD 3: Sips of water were allowed	Esophagectomy	Patients with esophageal cancer (primarily ESCC or EAC, some high-grade dysplasia)	Duration of hospital stay, postoperative complications, atrial fibrillation, urinary tract infection, urinary retention, pneumonia, anastomotic leak, effusion drainage, respiratory failure, mortality (30-day)	1 month
Pan 2014 [36]	Retrospective cohort	Single-center	China	80	FTS vs. SOC	POD 2: Oral feeding with stepwise progression POD 6: Early EN	POD 2	POD 2: EN started POD 6: Initiation of oral feeding with clear liquids	MIE	Patients aged 25–85 years with biopsy-confirmed esophageal cancer	Fluid transfusion volume, time to first flatus, postoperative hospital stay duration, mortality (in-hospital or within 30 days)	1 month
Speicher 2018 [37]	Retrospective cohort	Single-center	USA	203	EOF vs. LOF	-	POD 3	POD 3: NJ tube feeding POD 15: Initiation of oral feeding	THE with CEGA	Patients with esophageal malignancies (EAC, ESCC, neuroendocrine/small cell), Barrett’s esophagus with high-grade dysplasia, achalasia, or GERD/stricture	Anastomotic leak rate, anastomotic stricture rate, length of postoperative hospital stay, 30-day mortality, 90-day mortality, overall survival (1-, 3-, 5-year rates)	30 months
Suresh 2000 [38]	RCT	Single-center	India	35	EOF vs. SOC	POD 3: Feeding with water, progressing to liquids and semi-solids	POD 3	POD 5: Initiation of oral feeding, test feeding with water, progressing to liquids and semi-solids	Cervical esophagogastrostomy	Patients represented by benign conditions (corrosive stricture, achalasia) or malignant diseases (squamous-cell carcinoma, adenocarcinoma)	Anastomotic leak detected by gastrografin study, clinical anastomotic leak, timing of clinical leak onset, management of leaks	-
Tanishima 2021 [39]	Retrospective cohort	Single-center	Japan	119	SOC vs ER	POD 1: Physical rehabilitation POD 2: Ice cube POD 4: Soft fractionated diet and EN POD 12: Planned discharge	POD 2	POD 1: NG tube removed POD 6: Physiotherapy started POD 7: EN started POD 8: Soft diet POD 17: Planned discharge	MIMCE	Patients with esophageal cancer	Time to extubation, postoperative use of catecholamine, length of ICU stay, time to ambulation, time to oral feeding, time to flatus, time to defecation, CRP levels (PODs 1, 3, 7, and 10), length of hospital stay	1 Month
Zhang 2017 [40]	RCT	Single-center	China	94	Observation vs. control	POD 1: Start the feeding and mobilization POD 4: Combined intravenous nutrition, tapered if oral feeding was sufficient	POD 1	POD 0–7: Fluid infusion	Esophagectomy	Patients with esophageal cancer	Intraoperative blood loss, number of dissected lymph nodes, operation time, Postoperative hospital stay, cost of hospitalization, postoperative readmission rate (at 3 months)	3 months

**Abbreviations**: Early oral feeding (EOF); late oral feeding (LOF); direct oral feeding (DOF); standard of care (SOC); postoperative day (POD); nasogastric (NG); enteral nutrition (EN); Enhanced Recovery After Surgery (ERAS); nasojejunal (NJ); intravenous (IV); minimally invasive esophagectomy (MIE); minimally invasive Ivor Lewis esophagectomy (MIE-IL); transhiatal esophagectomy (THE); transthoracic esophagectomy (TTE); cervical esophagogastric anastomosis (CEGA); minimally invasive McKeown esophagectomy (MIMCE); American Society of Anesthesiologists (ASA); Body Mass Index (BMI); esophageal squamous cell carcinoma (ESCC); esophageal adenocarcinoma (EAC); gastroesophageal junction (GEJ); enhanced recovery protocol (ERP); enhanced recovery (ER); fast-track surgery (FTS); Intensive Care Unit (ICU); hemoglobin (Hb); albumin (ALB); prealbumin (PA); immunoglobulin A (IgA); immunoglobulin G (IgG); immunoglobulin M (IgM); C-reactive protein (CRP); interleukin-6 (IL-6); interleukin-8 (IL-8); tumor necrosis factor-alpha (TNF-α); monocyte chemoattractant protein-1 (MCP-1); Gastrointestinal Symptom Rating Scale (GSRS); brain–gut peptide (BGP); transferrin (TF); nil per os (NPO); gastroesophageal reflux disease (GERD); recurrent laryngeal nerve (RLN).

**Table 2:** Baseline characteristics of included studies.

Study ID	Follow-Up (Months)	Arm	N	Age (y) Mean ± SD	Male N (%)	BMI (kg/m^2^) Mean ± SD	Comorbidities			Smoking N (%)	Alcohol N (%)	Drug Hx		Pre-Op Labs				Tumor Thoracic Location Upper/Middle/Lower/Multiple	Pathological Type/Histology ESCC/EAC/Other	Differentiation Poor/Moderate/High	Duration of Surgery (min) Mean ± SD	Blood Loss During Surgery (mL) Mean ± SD	Anastomosis Method		Lymphadenectomy 3-Field/2-Field	Number of Excised LN Mean ± SD
							Cardiac N (%)	DM N (%)	Pulmonary N (%)			Chemotherapy N (%)	Radiotherapy N (%)	Hb (g/dL) Mean ± SD	ALB (g/dL) Mean ± SD	CRP (mg/L) Mean ± SD	TF (mg/dL) Mean ± SD						Side-to-Side Stapled N (%)	End-to-End Hand Sewn N (%)		
Berkelmans 2020 [10]	6 months	EOF	65	64.7 ± 8.34	56 (86.2)	25.9 ± 4.6	8 (12.3)	9 (13.8)	8 (12.3)	-	-	57 (87.7)	58 (89.23)	-	-	-	-	-	-	-	-	-	49 (75.4)	16 (24.6)	-	-
		LOF	67	65.3 ± 6.8	58 (86.6)	26.3 ± 3.4	17 (25.4)	6 (8.9)	7 (10.4)	-	-	64 (95.5)	63 (94.03)	-	-	-	-	-	-	-	-	-	49 (73.13)	18 (26.87)	-	-
Geraedts 2023 [11]	60 months	EOF	63	65.00 ± 8.35	54 (85.7)	26.00 ± 4.55	8 (12.7)	8 (12.7)	8 (12.7)	-	-	56 (88.9)	-	-	-	-	-	-	9/54/0	-	-	-	-	-	0/63	23.67 ± 9.10
		LOF	66	65.33 ± 6.82	57 (86.4)	26.33 ± 3.79	17 (25.8)	6 (9.1)	7 (10.6)	-	-	63 (95.5)	-	-	-	-	-	-	12/54/0	-	-	-	-	-	0/66	22.00 ± 7.58
Pattamatta 2021 [22]	6 months	EOF	47	-	-	-	-	-	-	-	-	-	-	-	-	-	-	-	-	-	-	-	-	-	-	-
		LOF	48	-	-	-	-	-	-	-	-	-	-	-	-	-	-	-	-	-	-	-	-	-	-	-
Fransen 2022 [12]	1 month	EOF	85	64.33 ± 9.05	68 (80.0)	25.77 ± 3.85	10 (11.8)	8 (9.4)	11 (12.9)	-	-	76 (89.4)	76 (89.4)	-	-	-	-	-	-	-	242.67 ± 33.94	133.33 ± 75.41	-	-	-	-
		LOF	111	67.33 ± 9.76	89 (80.2)	25.50 ± 3.23	30 (27.0)	9 (8.1)	18 (16.2)	-	-	103 (92.8)	100 (90.1)	-	-	-	-	-	-	-	243.33 ± 38.31	133.33 ± 75.11	-	-	-	18.62 ± 11.16
Jafarian 2005 [18]	-	EOF	13	63 ± 13.39	8 (61.5)	-	-	-	-	-	-	-	-	-	3.73	-	-	0/1/12/0	12/1/0	-	-	-	0 (0)	10 (76.9)	-	-
		LOF	10	54.9	5 (50)	-	-	-	-	-	-	-	-	-	3.64	-	-	0/2/8/0	9/1/0	-	-	-	0 (0)	10 (100)	-	-
Jiang 2021 [16]	1 month	EOF	67	63.37 ± 7.49	50 (74.6)	22.69 ± 2.19	-	67 (100)	-	41 (61.2)	44 (65.7)	7 (10.4)	-	13.18 ± 1.97	4.27 ± 0.43	2.86 ± 3.37	212 ± 36	4/45/18/0	67/0/0	18/40/9		227.62 ± 50.26	-	-	-	
		LOF	54	63.74 ± 7.22	41 (75.9)	23.12 ± 2.61	-	54 (100)	-	31 (57.4)	36 (66.7)	5 (9.3)	-	13.32 ± 2.44	4.32 ± 0.47	3.48 ± 5.72	206 ± 22	6/29/19/0	67/0/0	18/26/10		239.81 ± 55.83	-	-	-	20.34 ± 9.86
Li 2021 [19]	-	EOF	87	62.4 ± 7.34	64 (73.6)	22.03 ± 2.56	8 (9.2)	5 (5.7)	18 (20.7)	-	-	5 (5.7)	-	13.07 ± 2.08	4.01 ± 0.40	-	-	11/52/24	83/4/0	-	-	-	76 (87.4)	11 (12.6)	-	-
		LOF	92	62.7 ± 9.60	68 (73.9)	22.37 ± 3.12	11 (12.0)	8 (8.7)	23 (25.0)	-	-	8 (8.7)	-	12.88 ± 1.93	3.97 ± 0.42	-	-	9/68/15	88/4/0	-	-	-	79 (85.9)	13 (14.1)	-	-
Liao 2020 [20]	Until discharge	EOF	21	57 ± 8.2	14 (66.7)	21.76 ± 1.32	-	-	-	-	-	0 (0)	0 (0)	11.41 ± 0.84	3.83 ± 0.36	4.48 ± 1.26	231 ± 39	0/21 (Middle or lower)/0	-	-	235.0 ± 21.6	115.0 ± 53.4	0 (0)	21 (100)	-	-
		LOF	20	58 ± 9.8	13 (65)	21.03 ± 1.23	-	-	-	-	-	0 (0)	0 (0)	11.87 ± 0.98	3.75 ± 0.38	4.61 ± 1.29	240 ± 46	0/20 (Middle or lower)/0	-	-	228.3 ± 19.0	103.3 ± 48.2		20 (100)	-	-
Zadeh 2015 [21]	-	EOF	54	64.2 ± 8.2	29 (53.7)	28.4 ± 2.1	-	-	-	-	-	12 (22.2)	-	-	-	-	-	2/15/16/21	-	10/9/31	-	-	-	-	-	-
		LOF	55	66.4 ± 7.7	29 (52.7)	29 ± 2.3	-	-	-	-	-	14 (25.5)	-	-	-	-	-	2/16/15/22	-	10/11/30	-	-	-	-	-	-
Shoar 2016 [26]	-	EOF	72	61.4 ± 10.3	35 (48.6)	-	-	-	-	-	-	-	-	-	-	-	-	1/19/20/-	-	-	-	-	0 (0)	72 (100)	-	-
		LOF	108	61.6 ± 11.8	55 (50.9)	-	-	-	-	-	-	-	-	-	-	-	-	0/23/25/-	-	-	-	-	0 (0)	108 (100)	-	-
Sun 2015 [9]	-	EOF	68	61.1 ± 8.4	43 (63.2)	-	-	-	-	-	-	0 (0)	0 (0)	-	3.46 ± 0.24	-	-	15/28/25/0	68/0/0	18/21/29	-	-	0 (0)	68 (100)	0/68	-
		LOF	65	60.3 ± 7.0	44 (67.7)	-	-	-	-	-	-	0 (0)	0 (0)	-	3.54 ± 0.29	-	-	16/29/20/0	65/0/0	17/30/18	-	-	0 (0)	65 (100)	0/65	-
Sun 2018 [23]	6 months	EOF	140	63.00 ± 7.49	92 (65.7)	23.60 ± 3	-	-	-	-	-	36 (25.7)	2 (1.4)	-	4.38 ± 0.35	-	-	25/59/54/2	132/2/6	33/56/43	186.00 ± 31.46	-	0 (0)	140 (100)	4/136	27.67 ± 12.73
		LOF	140	63.33 ± 8.24	103 (73.6)	23.60 ± 3.15	-	-	-	-	-	47 (33.6)	2 (1.4)	-	4.35 ± 0.47	-	-	20/76/41/3	133/4/3	42/55/36	187.33 ± 29.96	-	0 (0)	140 (100)	5/135	27.67 ± 9.74
Sun 2019 [24]	-	EOF	46	61.33 ± 12.24	28 (60.9)	23.57 ± 3.06	-	-	-	-	-	14 (30.4)	-	-	4.34 ± 0.35	-	-	5/24/16/1	45/0/1	16/20/6	215.00 ± 45.15	120.00 ± 61.22	0 (0)	46 (100)	2/44	28.33 ± 12.24
		LOF	40	63.33 ± 8.46	24 (60)	23.60 ± 3.23	-	-	-	-	-	10 (25)	-	-	4.39 ± 0.46	-	-	4/20/15/1	38/2/0	14/16/7	207.00 ± 43.06	113.33 ± 53.83	0 (0)	40 (100)	2/38	28.33 ± 10
Weijs 2016 [25]	-	EOF	50	66.00 ± 8.40	42 (84)	-	16 (32)	10 (20)	15 (30)	7 (14)	-	49 (98)	48 (96)	-	-	-	-	-	4/45/1	-	252.33 ± 61.06	133.33 ± 76.33	-	-	-	-
		LOF	50	64.67 ± 9.92	40 (80)	-	15 (30)	8 (16)	12 (24)	11 (22)	-	48 (96)	41 (82)	-	-	-	-	-	1/48/1	-	325.33 ± 78.62	271.00 ± 305.32	-	-	-	-
Yang 2022 [8]	1 month	EOF	50	60.62 ± 6.74	32 (64)	22.42 ± 1.30	-	-	-	-	-	-	-	-	-	-	-	8/30/12/0	-	-	198.32 ± 19.27	116.10 ± 27.43	-	-	-	-
		LOF	50	60.28 ± 6.61	28 (56)	21.97 ± 1.25	-	-	-	-	-	-	-	-	-	-	-	8/30/12/0	-	-	198.12 ± 35.99	108.10 ± 32.37	-	-	-	-
Berkelmans 2017 [27]	26.5 months	EOF	50	66.00 ± 8.40	43 (86)	26.10 ± 3.51	-	-	-	-	-	1 (2)	48 (96)	-	-	-	-	0/6/36/-	45/4/0	-	-	-	-	-	-	-
	35.8 months	LOF	64	65.00 ± 9.86	55 (86)	25.30 ± 2.88	-	-	-	-	-	7 (11)	54 (84)	-	-	-	-	1/4/38/-	61/2/0	-	-	-	-	-	-	-
Bolton 2013 [28]	-	EOF	87	61.8 ± 10	82 (83.7)	-	-	-	-	-	-	44 (44.4)	-	-	-	-	-	-	-	-	-	-	-	-	-	-
		LOF	33		16 (76.2)	-	-	-	-	-	-	10 (47.6)	-	-	-	-	-	-	-	-	-	-	-	-	-	-
Chen 2016 [29]	1 month	EOF	128	56.43 ± 13.28	103 (80.5)	22.53 ± 2.85	-	-	-	-	-	68 (53.1)	27 (21.1)	-	-	4.97 ± 1.33	-	12/73/43/0	116/7/5	-	168.98 ± 30.62	302.54 ± 88.48	-	-	-	-
		LOF	132	55.72 ± 10.34	106 (80.3)	22.89 ± 2.56	-	-	-	-	-	70 (53.0)	25 (18.9)	-	-	4.85 ± 1.43	-	14/76/42/0	121/8/3	-	172.33 ± 24.67	312.33 ± 76.73	-	-	-	-
Chen 2022 [30]	-	EOF	63	62.67 ± 6.83	48 (76.2)	22.63 ± 2.88	-	-	-	-	-	17 (27.0)	-	-	4.30 ± 0.31	-	249 ± 68.3	11/26/26/0	63/0/0	33/25/5	195.00 ± 26.55	38.33 ± 15.17	0 (0)	63 (100)	-	-
		LOF	47	64.00 ± 7.65	32 (68.1)	23.47 ± 4.51	-	-	-	-	-	14 (29.8)	-	-	4.31 ± 0.32	-	241 ± 56.6	9/18/20/0	47/0/0	21/23/3	200.00 ± 34.41	43.33 ± 22.94	0 (0)	47 (100)	-	-
Ding 2020 [31]	-	EOF	42	58.47 ± 6.25	22 (52.4)	-	-	-	-	-	-	6 (14.3)	-	8.15 ± 1.05	2.15 ± 0.43	-	240 ± 98	8/18/16/0	38/4/0	-	195.48 ± 36.44	49.07 ± 19.32	-	-	-	-
		LOF	58	60.68 ± 5.42	22 (52.4)	-	-	-	-	-	-	7 (12.1)	-	8.27 ± 0.97	2.23 ± 0.42	-	240 ± 98	11/30/17/0	50/8/0	-	201.45 ± 40.33	48.27 ± 16.45	-	-	-	-
Eberhard 2017 [32]	2 months	EOF	117	64	94 (80)	29	16 (14)	25 (21)	2 (2)	34 (29)	10 (8)	99 (85)	-	-		-	-	-	0/117/0	-	-	-	-	-	-	-
		LOF	90	64	74 (82)	27	6 (6)	9 (10)	6 (7)	25 (28)	13 (15)	72 (80)	-	-		-	-	-	0/90/0	-	-	-	-	-	-	-
Giacopuzzi 2017 [33]	3 months	EOF	22	61.00 ± 27.74	18 (81.8)	26.100 ± 5.237	-	-	-	19 (86.3)	10 (45.4)	22 (100)	18 (81.8)	-		-	-	0/6/16/0	7/14/1	-	-	-	-	-	-	-
		LOF	17	61.33 ± 25.06	15 (88.2)	25.900 ± 5.400	-	-	-	12 (70.5)	8 (47.1)	16 (94.1)	13 (76.4)	-		-	-	1/11/5/0	8/9/0	-	-	-	-	-	-	-
Hao 2023 [34]	3 months	EOF	112	62.4 ± 4.8	84 (75)	24.0 ± 2.2	-	-	-	-	-	112 (100)	-	-		-	-	21/67/24/0	107/2/3	38/35/38	246.7 ± 27.8	77.5 ± 25.0	112 (100)	0 (0)	-	32.8 ± 7.1
		LOF	69	61.9 ± 4.9	51 (74)	24.9 ± 1.6	-	-	-	-	-	69 (100)	-	-		-	-	16/44/9/0	65/1/3	29/17/27	252.3 ± 31.8	79.2 ± 30.0	69 (100)	0 (0)	-	33.2 ± 6.8
Nevo 2021 [35]	1 month	EOF	69	67 ± 10	59 (85)	25.9 ± 4.7	-	-	-	-	-	-	-	-		-	-	-	8/61/0	-	280 ± 59	-	0 (0)	69 (100)	2/60	-
		LOF	59	64 ± 10	47 (80)	26.7 ± 6.1	-	-	-	-	-	-	-	-		-	-	-	4/51/4	-	269 ± 83	-	0 (0)	59 (100)	-	-
Pan 2014 [36]	1 month	EOF	40	65.0 ± 6.7	29 (72.5)	-	-	-	-	-	-	-	-	-	4.03 ± 0.41	-	-	3/33/4/0	39/0/1	-	-	-	0 (0)	40 (100)	-	-
		LOF	40	62.9 ± 5.9	29 (72.5)	-	-	-	-	-	-	-	-	-	4.04 ± 0.36	-	-	1/36/3/0	40/0/0	-	-	-	0 (0)	40 (100)	-	-
Speicher 2018 [37]	30 months	EOF	83	62 ± 11.2	65 (78.3)	26.5 ± 6.5	-	-	-	-	-	42 (50.6)	-	-		-	-	-	7/61/15	-	212 ± 74.7	269 ± 156.4	83 (100)	0 (0)	-	-
		LOF	120	63 ± 10.6	101 (84.2)	27.6 ± 6.1	-	-	-	-	-	83 (69.2)	-	-		-	-	-	14/98/8	-	212 ± 48.1	244 ± 141.9	120 (100)	0 (0)	-	-
Suresh 2000 [38]	-	EOF	17	42.9	11 (64.7)	-	5 (29.4)	2 (11.8)	0 (0)	-	-	-	11 (64.7)	12.44	4.12	-	-	-	11/3/0	-	-	563.15	-	-	-	-
		LOF	16	55.68	11 (68.8)	-	4 (25.0)	1 (6.3)	1 (6.3)	-	-	-	9 (56.3)	12.63	4	-	-	-	10/5/0	-	-	593.15	-	-	-	-
Tanishima 2021 [39]	1 month	EOF	42	68.4 ± 6.0	33 (78.6)	21.8 ± 3.3	-	-	-	-	-	26 (61.9)	-	-	4.0 ± 0.4	-	26.4 (4.4)	-	39/3/0	-	550.4 ± 71.3	177.6 ± 152.3	0 (0)	42 (100)	32/10	-
		LOF	42	68.3 ± 5.8	36 (85.7)	22.1 ± 3.6	-	-	-	-	-	25 (59.5)	-	-	3.9 ± 0.4	-	26.3 (5.4)	-	41/1/0	-	549.4 ± 74.8	188.6 ± 131.3	0 (0)	42 (100)	37/5	-
Zhang 2017 [40]	1 month	EOF	47	56.03 ± 6.56	25 (53.2)	-	-	-	-	-	-	-	-	-		-	-	-	-	-	264.73 ± 8.05	56.43 ± 11.63	-	-	-	27.23 ± 1.56
		LOF	47	55.86 ± 6.43	28 (59.6)	-	-	-	-	-	-	-	-	-		-	-	-	-	-	265.24 ± 8.37	56.43 ± 11.63	-	-	-	27.56 ± 2.47

Abbreviations: albumin (ALB), C-reactive protein (CRP), transferrin (TF).

## Data Availability

All data generated or analyzed during this study is presented in this article.

## Supplementary Materials

The following supporting information can be downloaded at: https://www.mdpi.com/article/10.3390/jpm15070317/s1, Supplementary Table S1: Detailed literature search strategy and number of results for each electronic database; Supplementary Table S2: Quality assessment of included observational studies using the Newcastle-Ottawa Scale (NOS); Supplementary Table S3: GRADE (Grading of Recommendations, Assessment, Development, and Evaluation) assessment of the certainty of evidence for the included outcomes.

## Abbreviations

ALB, albumin; ARDS, acute respiratory distress syndrome; ASA, American Society of Anesthesiologists; BGP, brain–gut peptide; BMI, Body Mass Index; CEGA, cervical esophagogastric anastomosis; CI, confidence interval; CRP, C-reactive protein; DOF, direct oral feeding; EAC, esophageal adenocarcinoma; EN, enteral nutrition; EOF, early oral feeding; EORTC, European Organization for Research and Treatment of Cancer; ER, enhanced recovery; ERAS, Enhanced Recovery After Surgery; ERP, enhanced recovery protocol; ESCC, esophageal squamous-cell carcinoma; FTS, fast-track surgery; GEJ, gastroesophageal junction; GERD, gastroesophageal reflux disease; Hb, hemoglobin; ICU, Intensive Care Unit; LOF, late oral feeding; LOS, length of hospital stay; MCP-1, monocyte chemoattractant protein-1; MIE, minimally invasive esophagectomy; MIMCE, minimally invasive McKeown esophagectomy; NG, nasogastric; NJ, nasojejunal; NOS, Newcastle–Ottawa Scale; NPO, nil per os (nil-by-mouth); POD, postoperative day; QoL, quality of life; QLQ-C30, Quality of Life Questionnaire—Core 30; QLQ-OES18/OG25, Quality of Life Questionnaire—Esophageal Module 18/25; THE, transhiatal esophagectomy; TTE, transthoracic esophagectomy.
