# Supplementary material for: Early vs. Late Oral Feeding After Surgery for Patients with Esophageal Malignancy: A Systematic Review and Meta-Analysis of Postoperative Clinical Outcomes and Quality of Life
Source: J Pers Med. 2025 Jul 15;15(7):317. doi: 10.3390/jpm15070317 (PMC12299261; doi:10.3390/jpm15070317)
Supplement: Supplementary file 1 [file jpm-15-00317-s001.zip › Supplementary Table 1 (Modeified).pdf]

Date of Search

28 February 2025

Search strategy

Database	Search Query	Number of results
PubMed	(Oesophagectomy OR Esophagectomy OR “Esophageal resection” OR “Esophagus resection” OR “Esophagus excision” OR “Esophageal excision” OR “Esophageal removal” OR “Esophagus removal” OR Esophagogastrectomy OR “Esophageal reconstruction” OR “Esophagus reconstruction”) OR "Esophagectomy"[Mesh]<br>AND<br>(“Oral Feed*” OR “Food Intake” OR “Per os feeding” OR “PO feeding” OR “Oral ingestion” OR “Oral intake” OR “Deglutition”)	1393
Scopus	(Oesophagectomy OR Esophagectomy OR “Esophageal resection” OR “Esophagus resection” OR “Esophagus excision” OR “Esophageal excision” OR “Esophageal removal” OR “Esophagus removal” OR Esophagogastrectomy OR “Esophageal reconstruction” OR “Esophagus reconstruction”)<br>AND<br>(“Oral Feed*” OR “Food Intake” OR “Per os feeding” OR “PO feeding” OR “Oral ingestion” OR “Oral intake” OR “Deglutition”)	1298
Web of science (WoS)	(ALL=(Oesophagectomy) OR ALL=(Esophagectomy) OR ALL=(Esophageal resection) OR ALL=(Esophagus resection) OR ALL=(Esophagus excision) OR ALL=(Esophageal excision) OR ALL=(Esophageal removal) OR ALL=(Esophagus removal) OR ALL=(Esophagogastrectomy) OR ALL=(Esophageal reconstruction) OR ALL=(Esophagus reconstruction))<br>AND<br>(ALL=(Oral Feed) OR ALL=(Food Intake) OR ALL=(Per os feeding) OR ALL=(PO feeding) OR ALL=(Oral ingestion) OR ALL=(Oral intake) OR ALL=(Deglutition))	832
Cochrane CENTRAL	(Oesophagectomy OR Esophagectomy OR “Esophageal resection” OR “Esophagus resection” OR “Esophagus excision” OR “Esophageal excision” OR “Esophageal removal” OR “Esophagus removal” OR Esophagogastrectomy OR “Esophageal reconstruction” OR “Esophagus reconstruction”)<br>AND<br>(“Oral Feed*” OR “Food Intake” OR “Per os feeding” OR “PO feeding” OR “Oral ingestion” OR “Oral intake” OR “Deglutition”)	101
EMBASE	(Oesophagectomy OR Esophagectomy OR “Esophageal resection” OR “Esophagus resection” OR “Esophagus excision” OR “Esophageal excision” OR “Esophageal removal” OR “Esophagus removal” OR Esophagogastrectomy OR “Esophageal reconstruction” OR “Esophagus reconstruction”)<br>AND<br>(“Oral Feed*” OR “Food Intake” OR “Per os feeding” OR “PO feeding” OR “Oral ingestion” OR “Oral intake” OR “Deglutition”)	2392

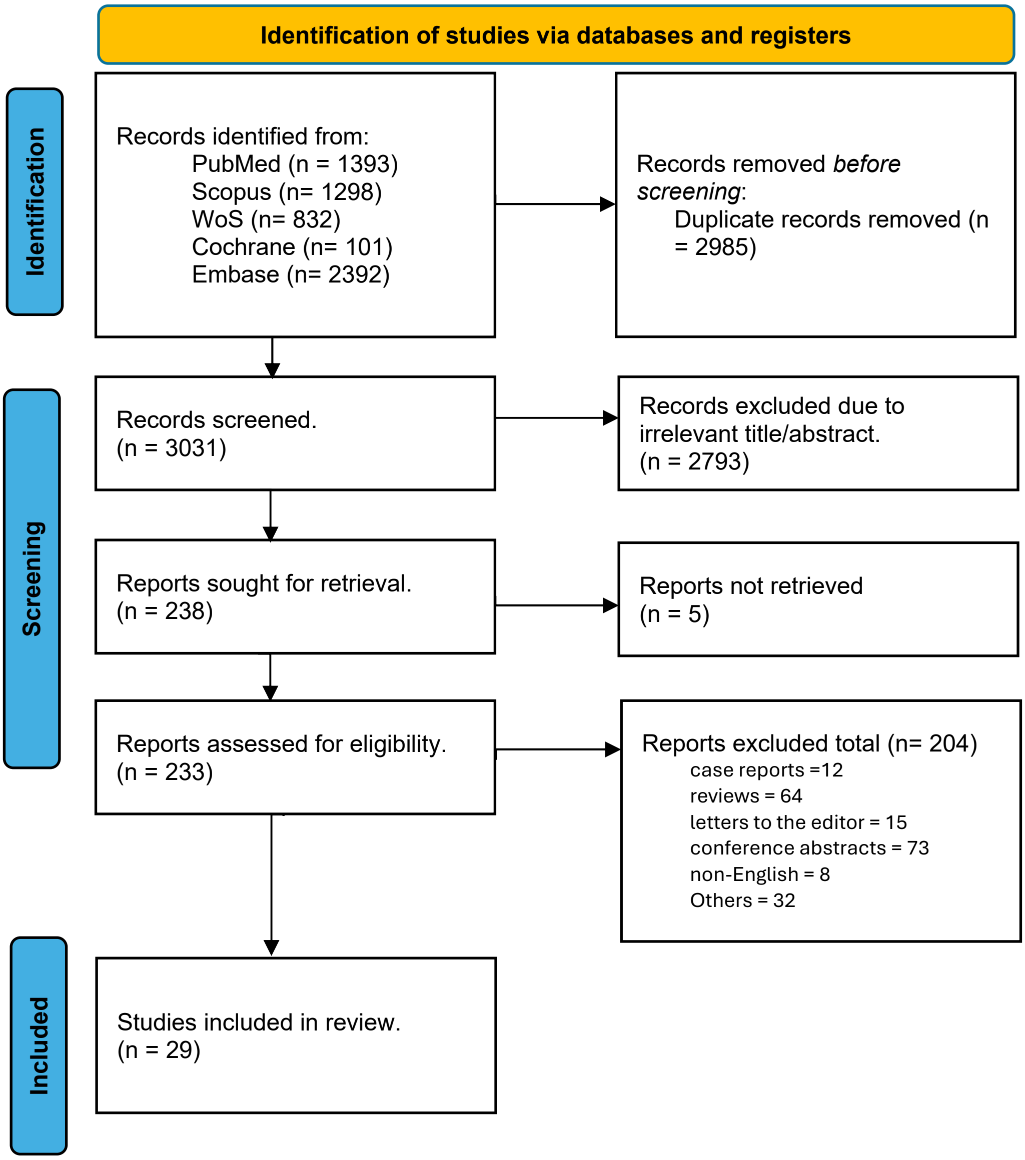

From: Page MJ, McKenzie JE, Bossuyt PM, Boutron I, Hoffmann TC, Mulrow CD, et al. The PRISMA 2020 statement: an updated guideline for reporting systematic reviews. BMJ 2021;372:n71. doi: 10.1136/bmj.n71
