# Supplementary material for: Early vs. Late Oral Feeding After Surgery for Patients with Esophageal Malignancy: A Systematic Review and Meta-Analysis of Postoperative Clinical Outcomes and Quality of Life
Source: J Pers Med. 2025 Jul 15;15(7):317. doi: 10.3390/jpm15070317 (PMC12299261; doi:10.3390/jpm15070317)
Supplement: Supplementary file 1 [file jpm-15-00317-s001.zip › Supplementary Table 2 NOS.pdf]

Cohort studies													
Baseline					Selection				Comparability	Outcome			Quality Score
**Study Title**	**Study Design (Prospective or retrospective)**	**mean follow up**	**Sample (n) (EOF/LOF)**	**Age at baseline mean (Year) (EOF/LOF)**	**Representativeness of the exposed cohort**	**Selection of the non-exposed cohort**	**Ascertainment of exposure**	**Demonstration that outcome of interest was not present at start of study**	**Comparability of cohorts on the basis of the design or analysis**	**Assessment of outcome**	**Was follow-up long enough for outcomes to occur**	**Adequacy of follow up of cohorts**	
Fransen 2022	Prospective cohort	1 month	(85/111)	(64.33/67.33)	*	*	*	*	**	*	*	*	Good quality
Jafarian 2005	Prospective cohort	-	(13/10)	(63/54.9)	*	*	*	*	**				Moderate quality
Jiang 2021	Retrospective cohort	1 month	(67/54)	(63/63.74)	*	*	*	*	**	*	*	*	Good quality
Li 2021	Retrospective cohort	Until hospital discharge	(87/92)	(62.4/62.7)	*	*	*	*	**	*			Good quality
Sun 2015	Prospective cohort	Until hospital discharge	(68/65)	(61.1/60.3)	*	*	*	*	**	*			Good quality
Weijs 2016	prospective cohort	3 months	(50/50)	(66/68.4)	*	*	*	*	**	*	*	*	Good quality
Shoar 2016	prospective cohort	-	(72/108)	(61.4/61.6)	*	*	*	*	**	*			Good quality
Berkelmans 2017	Retrospective cohort	12 months	(50/64)	(66/65)	*	*	*	*	**	*	*		Good quality
Bolton 2013	Retrospective cohort	--	(87/33)	-	*	*	*	*		*			Moderate quality
Eberhard 2017	Retrospective cohort	2 months	(117/90)	(64/64)	*	*	*	*	*	*	*	*	Good quality
Giacopuzzi 2017	Prospective cohort	3 months	(22/17)	(61/61.3)	*	*	*	*	*	*	*	*	Good quality
Hao 2023	Retrospective cohort	3 months	(112/69)	(62.4/61.9)	*	*	*	*	**	*	*	*	Good quality
Nevo 2021	Retrospective cohort	1 months	(69/59)	(67/64)	*	*	*	*	*	*	*	*	Good quality
Pan 2014	Retrospective cohort	1 months	(40/40)	(65/62.9)	*	*	*	*	*	*	*	*	Good quality
Speicher 2018	Retrospective cohort	30 months	(83/120)	(62/63)	*	*	*	*	**	*	*	*	Good quality
Tanishima 2021	Retrospective cohort	3 months	(42/42)	(68.4/68.3)	*	*	*	*	**	*	*	*	Good quality

Table 2: NOS scale for observational studies.
