# Supplementary material for: Early vs. Late Oral Feeding After Surgery for Patients with Esophageal Malignancy: A Systematic Review and Meta-Analysis of Postoperative Clinical Outcomes and Quality of Life
Source: J Pers Med. 2025 Jul 15;15(7):317. doi: 10.3390/jpm15070317 (PMC12299261; doi:10.3390/jpm15070317)
Supplement: Supplementary file 1 [file jpm-15-00317-s001.zip › Supplementary Table 3 GRADE (New Supplement).pdf]

EOF compared to LOF for Esophagectomy

Bibliography:

Certainty assessment							Summary of findings				
Participants (studies) Follow-up	Risk of bias	Inconsistency	Indirectness	Imprecision	Publication bias	Overall certainty of evidence	Study event rates (%)		Relative effect (95% CI)	Anticipated absolute effects	
							With LOF	With EOF		Risk with LOF	Risk difference with EOF
**Anastomotic Leakage**											
3166 (24 RCTs)	serious[a]	not serious	serious[b]	serious[c]	none	⊕◯◯◯ Very low[a,b,c]	114/1581 (7.2%)	130/1585 (8.2%)	**RR 1.03** (0.80 to 1.33)	114/1581 (7.2%)	**2 more per 1,000** (from 14 fewer to 24 more)
**Respiratory affection**											
2486 (18 non-randomised studies)	serious[a]	not serious	serious[d]	not serious	none	⊕◯◯◯ Very low[a,d]	225/1221 (18.4%)	178/1265 (14.1%)	**RR 0.83** (0.69 to 1.00)	225/1221 (18.4%)	**31 fewer per 1,000** (from 57 fewer to 0 fewer)
**Any arrhytmia**											
1144 (7 non-randomised studies)	serious[a]	not serious	not serious	not serious	none	⊕◯◯◯ Very low[a]	40/540 (7.4%)	42/604 (7.0%)	**RR 0.91** (0.58 to 1.43)	40/540 (7.4%)	**7 fewer per 1,000** (from 31 fewer to 32 more)
**Hospital Readmission rate**											
882 (6 non-randomised studies)	not serious	not serious	not serious	serious	none	⊕◯◯◯ Very low	23/458 (5.0%)	-/424	**RR 1.20** (0.68 to 2.11)	23/458 (5.0%)	**10 more per 1,000** (from 16 fewer to 56 more)
**Mortality**											

EOF compared to LOF for Esophagectomy

Bibliography:

Certainty assessment							Summary of findings				
1510 (18 non-randomised studies)	serious[a]	not serious	not serious	serious	none	⨁◯◯◯ Very low[a]	6/772 (0.8%)	-/738	**RR 0.93** (0.36 to 2.40)	6/772 (0.8%)	**1 fewer per 1,000** (from 5 fewer to 11 more)

Hospital Stay

2767 (20 non-randomised studies)	serious[a]	serious[e]	very serious	very serious	none	⨁◯◯◯ Very low[a,e]	1373	1394	-	-	SMD **0.62 SD lower** (1.05 lower to 0.19 lower)

CI: confidence interval; **RR:** risk ratio; **SMD:** standardised mean difference

Explanations

a. some of the included studies results at low risk of bias or with some concerns

b. variability in the definition and timing of "early oral feeding" (EOF) and "late oral feeding" (LOF) across studies

c. A pooled estimate of the effect would have a wide 95% confidence interval

d. While the primary population is patients undergoing esophagectomy for cancer, some studies include specific subgroups, such as patients with type II diabetes, or focus on particular surgical approaches like minimally invasive esophagectomy (MIE) with intrathoracic anastomosis, or McKeown MIE

e. Heterogeneity I2 = 96.38%
